# A generative approach for semantic auditing of electronic health records

**DOI:** 10.1038/s41746-026-02809-w

**Published:** 2026-05-30

**Authors:** Irena Girshovitz, Atai Ambus, Moni Shahar, Ran Gilad-Bachrach

**Affiliations:** 1 School of Biomedical Engineering, Faculty of Engineering, Tel Aviv University, Tel Aviv, Israel; 2 AI and Data Science Center of Tel Aviv University (TAD), Tel Aviv, Israel; 3 Safra Center for Bioinformatics, Tel Aviv University, Tel Aviv, Israel

**Keywords:** Computational biology and bioinformatics, Health care, Mathematics and computing, Medical research

## Abstract

The reliability of clinical artificial intelligence (AI) depends on high-quality data, yet Electronic Health Records are often inconsistent with existing scientific knowledge. Current quality assessments are limited: they either focus on syntax or rely on labor-intensive manual rules to capture semantic nuances. To overcome these scalability barriers, we propose Medical Data Pecking, a methodology that adopts software unit testing principles for medical data validation. It introduces Semantic Data Coverage, employing Large Language Models to generate context-aware tests that “peck" for inconsistencies between observed data and epidemiological evidence. To demonstrate this methodology, we implemented a reference tool using a Retrieval-Augmented Generation architecture that synthesizes medical literature into executable code. When applied to three datasets, this implementation generated dozens of tests per cohort, identifying discrepancies between observed distributions and epidemiological priors. These discrepancies encompass both genuine data inconsistencies and expected cohort-selection effects. This work provides an initial framework for scalable semantic auditing, shifting assurance from manual rules to the generative and context-sensitive verification required for trustworthy AI.

## Introduction

Electronic health records (EHRs) are increasingly used in healthcare research^1–4^, in developing machine learning models for clinical use^5–7^ and in the analysis of patient populations, treatment outcomes, and healthcare trends^8^.

However, EHR data quality remained a concern. Weiskopf & Weng^9^ found most EHR-based studies relied on basic data-quality checks. In a recent survey, 92% of AI practitioners reported experiencing data quality issues causing negative downstream effects^10^. Kilkenny & Robinson^11^ emphasize that poor data quality undermines results, coining the phrase “Garbage in - Garbage out". Accordingly, data must be accurate, valid, complete, and available^11,12^.

In this work, we propose the adoption of quality assurance techniques from engineering disciplines to enhance process efficiency and ensure reliable and high-quality outcomes^13^. Specifically, in software engineering, unit tests are typically written prior to or immediately following code implementation^14^. Ideally, all execution paths are covered by unit tests. These local tests are designed to evaluate a specific segment of code to verify its correctness and are run frequently to identify potential bugs early and close to their origin. Consequently, automated test suites are commonly integrated into the Continuous Integration/Continuous Development (CI/CD) process^15^.

We argue that in data-driven applications, software engineering best practices should be applied not only to the codebase but also to the data itself, as errors in either can lead to system failures or misleading conclusions. Such practices should encompass all components of the application, including the data preprocessing pipeline, where issues may affect any variable, not just those detected by downstream statistical analyses. Faults in raw data or early-stage processing can propagate unnoticed and ultimately compromise research validity.

The evaluation of data quality should consider the context of the analysis with respect to populations, geographies, specific time frames, and medical conditions^16^. Existing quality assessment methods often neglect these contextual factors, focusing instead on syntactic completeness^17^.

Although concordance with external datasets and prior knowledge could bridge these gaps, most current tools omit this due to the complexity of manually defining rules for every possible clinical scenario^17^. We propose using Large Language Models (LLMs) to automate the creation of such rules for a given use case.

Recent breakthroughs in natural language processing, particularly the introduction of LLMs, are reshaping multiple domains, including medical data analysis. LLMs have been applied to automate data extraction^18^, recognize clinical phenotypes^19–21^, curate concept sets^22^, explore datasets^23^, predict clinical risk^24^, and manage time-sequenced events during data preprocessing^25^. Narayan et al.^26^ evaluated LLMs across a range of data-related tasks, demonstrating state-of-the-art performance in data imputation, integration and error detection, even without task-specific fine-tuning.

Building on these capabilities, we introduce the Medical Data Pecking approach. We propose Medical Data Pecking to add systematic semantic auditing to complement existing data validation tools. While Medical Data Pecking can perform baseline structural checks, its primary utility is the identification of Semantic Gaps—instances where data maintains structural validity yet lacks epidemiological consistency. We demonstrate this approach through a reference implementation that utilizes LLMs to dynamically generate unit tests tailored to the target population and dataset. Analogously to selective pecking behavior in birds, Medical Data Pecking scans structured health records to identify relevant data fields, flag inconsistencies, and exclude extraneous content. It applies the generated tests to the data, identifies potential issues, and reports untested fields to quantify the coverage of the test suite.

## Results

We applied MDPT across multiple datasets to evaluate whether generative semantic testing can identify discrepancies beyond those captured by syntactic validation.

### Validation of automated test generation

**Fig. 1:**
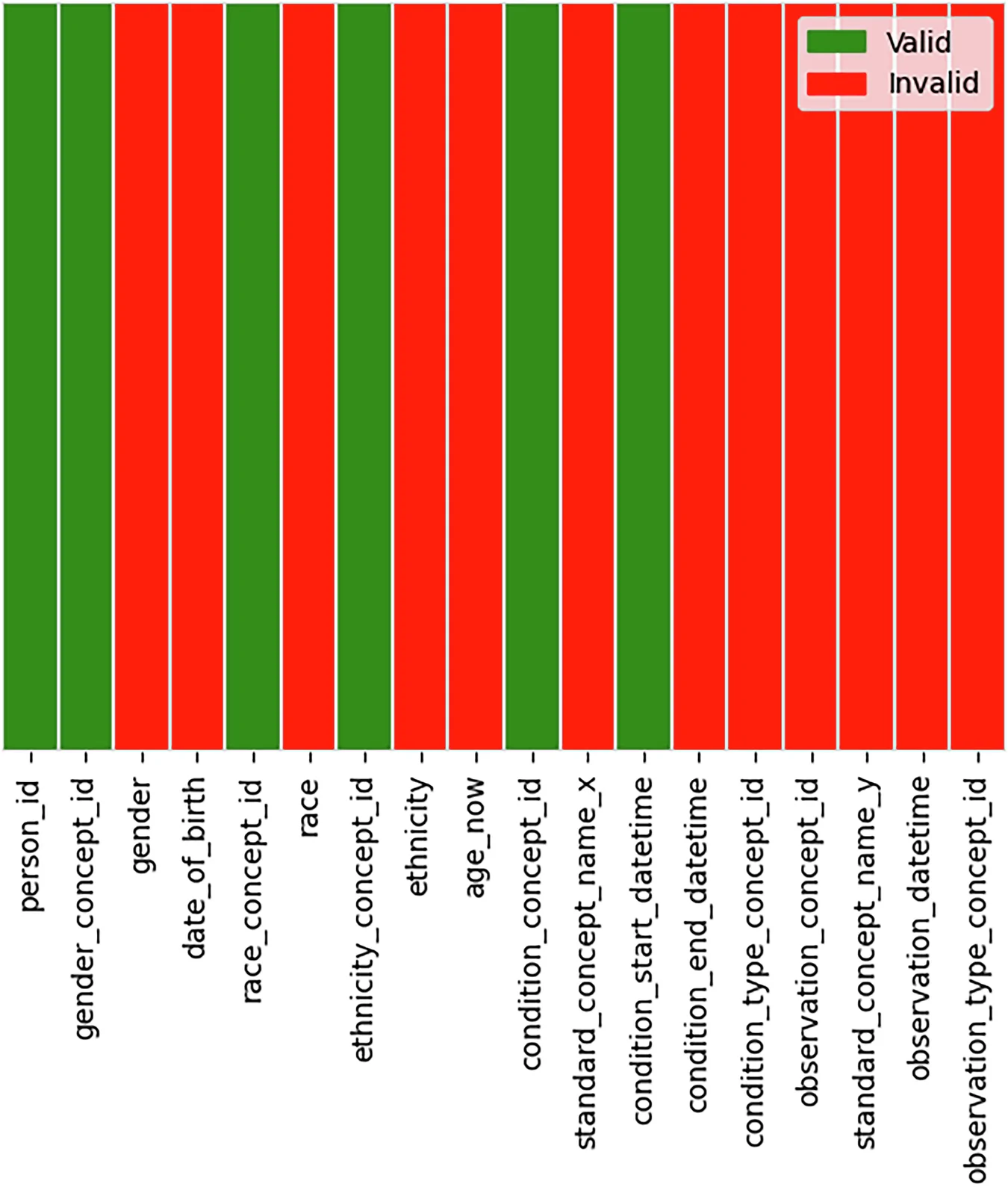
Semantic data coverage heatmap. This representative example illustrates the density of semantic validation across clinical variables. The dense red clusters (see annotations) highlight systemic Semantic Gaps, areas where data is syntactically present (gray in standard tools) but fails validation against epidemiological priors (red in MDPT).

To assess the reliability of the Medical Data Pecking framework in synthesizing semantic unit tests, we evaluated the quality of the test suites produced by its reference implementation, the Medical Data Pecking Tool (MDPT), across four clinical cohorts: Type 2 Diabetes (T2D) in All of Us (AoU), Chronic Kidney Disease in AoU (CKD), Hypertension in SyntheticMass (HTN), and Congestive Heart Failure in MIMIC-III (CHF). The framework autonomously generated between 55 and 73 unit tests per cohort (an example is provided in Fig. S2). The breadth of this coverage is visualized in Fig. 1, which displays the density of semantic validation across clinical variables, distinguishing between tested (green/red) and untested (gray) fields.

Across all datasets, the Auditor Agent preserved test instances through automated correction rather than simple exclusion. In the CHF cohort, which presented the highest initial alignment variance, the Auditor corrected 55.0% of the original tests to match clinical references, while only 10.0% were discarded. For further quantification of the Auditor Agent’s role in refining this initial pool see Table S4. Following the verification pass, the final refined test suites across all cohorts are documented in Table S5.

We evaluated the correctness of the literature-derived expected values against the source text and other published and publicly accessible sources in a single-reviewer curation process. In all suites, the reference validity ranged from 35% to 93% (Table S1). The lower bounds were mainly driven by drug prevalence tests, for which public data on pharmaceutical usage is often unavailable or fragmented. When removing drug prevalence tests, the validity of the semantic anchors increased to 39–98% (Table S7), with accurate information grounding present in up to 69% of the tests.

### Sensitivity to known biases (control validation)

Because real-world datasets lack a gold standard answer key for data quality, we validated the tool’s discernment using a positive control approach. To do this, we examined its performance on the AoU dataset, which is known to have specific recruitment skews^27^.

Without manual configuration, the MDPT framework correctly flagged demographic anomalies. Specifically, the generated semantic tests for gender and race distributions failed, accurately identifying the known over-representation of women and under-represented groups in the AoU cohort^27^. This serves as a positive control, confirming that the dynamic semantic testing paradigm is capable of autonomously detecting deviations from general population priors without explicit user programming.

### Uncovering semantic divergence

The primary contribution of the semantic testing paradigm is the detection of discrepancies that pass syntactic checks (valid codes) but fail epidemiological checks (plausible meaning). The framework identified substantial divergence across all tested datasets (Table S9). Importantly, these divergences do not imply that the datasets are unsuitable for research. Instead, they highlight factors that should be considered when evaluating whether a dataset is appropriate for a specific research question.

**Fig. 2:**
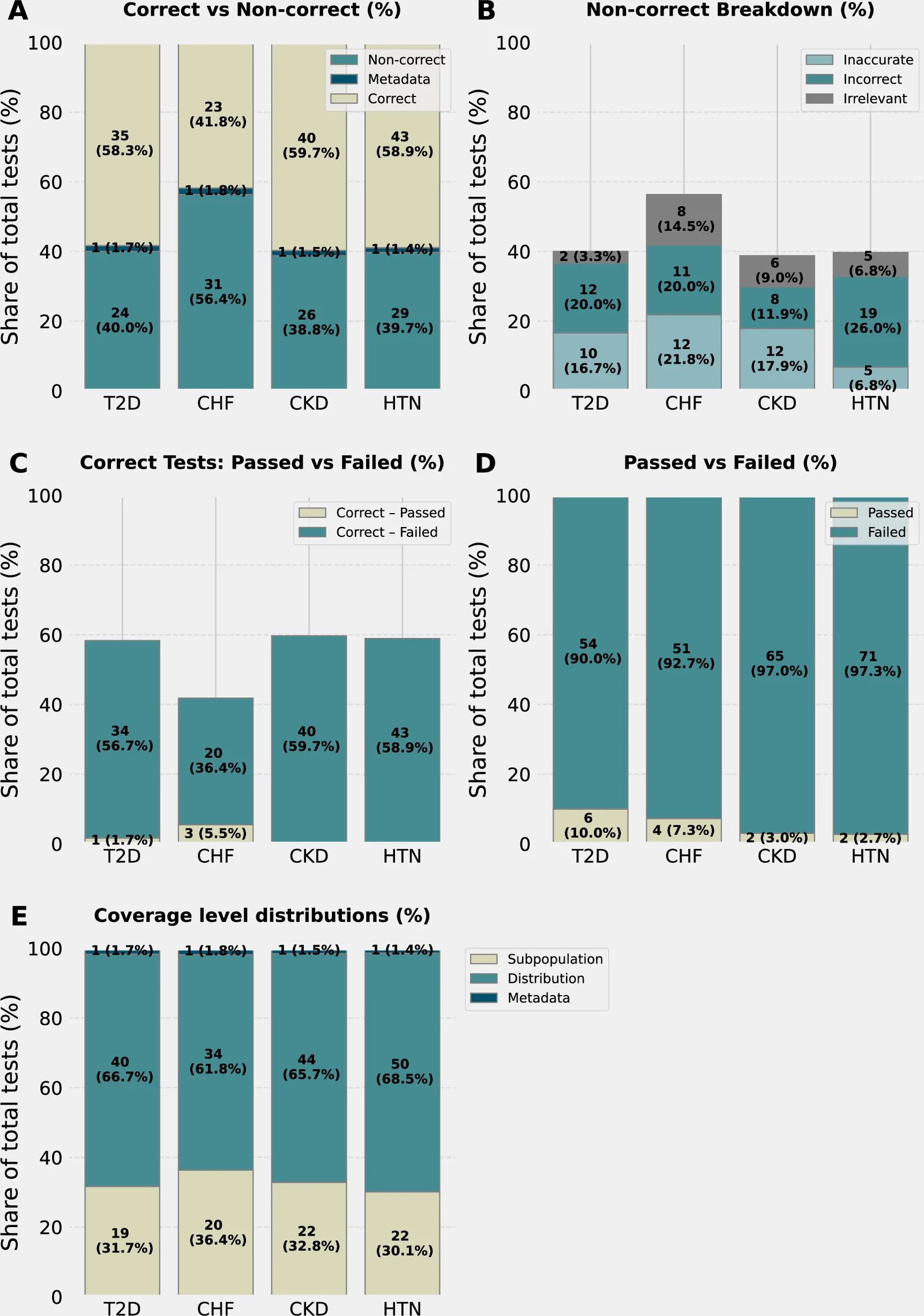
Quantifying the Semantic Gap. Evaluation summary of test suites generated by MDPT across four cohorts (T2D, CHF, CKD, HTN). **A**–**E** show quality metrics normalized by the number of generated tests per cohort. Detailed definition of these metrics can be found in S2 in the Supplementary Materials: **A** Proportion of tests with correct vs. non-correct reference values. **B** Breakdown of non-correct test outcomes by subtype, stratified by pass/fail status. **C** Distribution of correct test outcomes stratified by pass/fail status. **D** Overall proportion of passed vs. failed tests. **E** Distribution of tests across data coverage dimensions: subpopulation, distribution, and metadata levels.

As detailed in Fig. 2, our testing taxonomy is designed to capture semantic depth. Panel E illustrates that the vast majority of generated tests targeted the Distributional and Subpopulation (Contextual) semantic layers, while simple Metadata (syntactic) checks accounted for less than 2% of the test suite. The consequences of this deeper validation are visible in Panel D: across all cohorts, between 90% and 97% of these semantic tests failed. Panel C reports outcomes restricted to the ‘Correct’ test subset. This subset consists of instances where the Auditor Agent successfully verified the literature-derived reference values against grounded epidemiological evidence. As shown in Panel C, failure rates within this verified subset were 87.1% in the CHF cohort (36.4% failed vs. 5.5% passed), 97.3% in the T2D cohort (56.7% failed vs. 1.7% passed), and 100% in both the HTN and CKD cohorts.

In the AoU T2D cohort, 54 of the 60 generated tests failed. Although the data were syntactically complete, the framework revealed substantial discrepancies between the observed prevalence of comorbidities and expectations derived from the literature. Similarly, in the CKD cohort, 65 of the 67 tests failed.

In the MIMIC-III dataset evaluation for CHF, the framework detected a systematic formatting issue that rendered valid data semantically invisible. The tests failed due to a mismatch in ICD-9 coding standards (omission of decimal points). While standard syntactic checks validated these strings as non-null and allegedly correct, the semantic test, expecting a specific diagnosis prevalence, dropped to near zero, triggering a failure flag. This example shows that the framework can identify semantic discrepancies that may compromise downstream analysis. Although the omission of decimal points may appear to be a trivial formatting nuance, it represents a semantic break. Consequently, valid but dot-less ICD-9 codes were not captured by standard phenotype algorithms. MDPT detected this by identifying that the observed prevalence of the disease was inconsistent with epidemiological priors.

### Failure and robustness analysis

Beyond distributional deviations, we examined failures in which the observed prevalence of a disease, measurement, or drug prescription was 0%. These cases were analyzed separately because a zero observed value may indicate something different from ordinary statistical misalignment. Depending on the context, it may reflect a technical artifact, a cohort specification gap, or a mismatch between the queried concept and the dataset representation.

The pattern differed across cohorts. In the CHF cohort, zero-prevalence failures were primarily technical artifacts, mostly linked to the systematic omission of decimal points in ICD-9 codes. In the HTN cohort, these values were associated with gaps in the modeling of secondary comorbidities. In the CKD and T2D cohorts, they often reflected specification gaps during cohort construction, where relevant clinical items were overlooked in the initial cohort definition.

Across all cohorts, medication-related zero-prevalence failures frequently resulted from granularity mismatch. The LLM-generated tests targeted highly specific medication codes that did not align with the ingredient-level abstraction or the modeling choices of the target datasets. A list of all zero-prevalence failed tests is provided in Table S10.

**Fig. 3:**
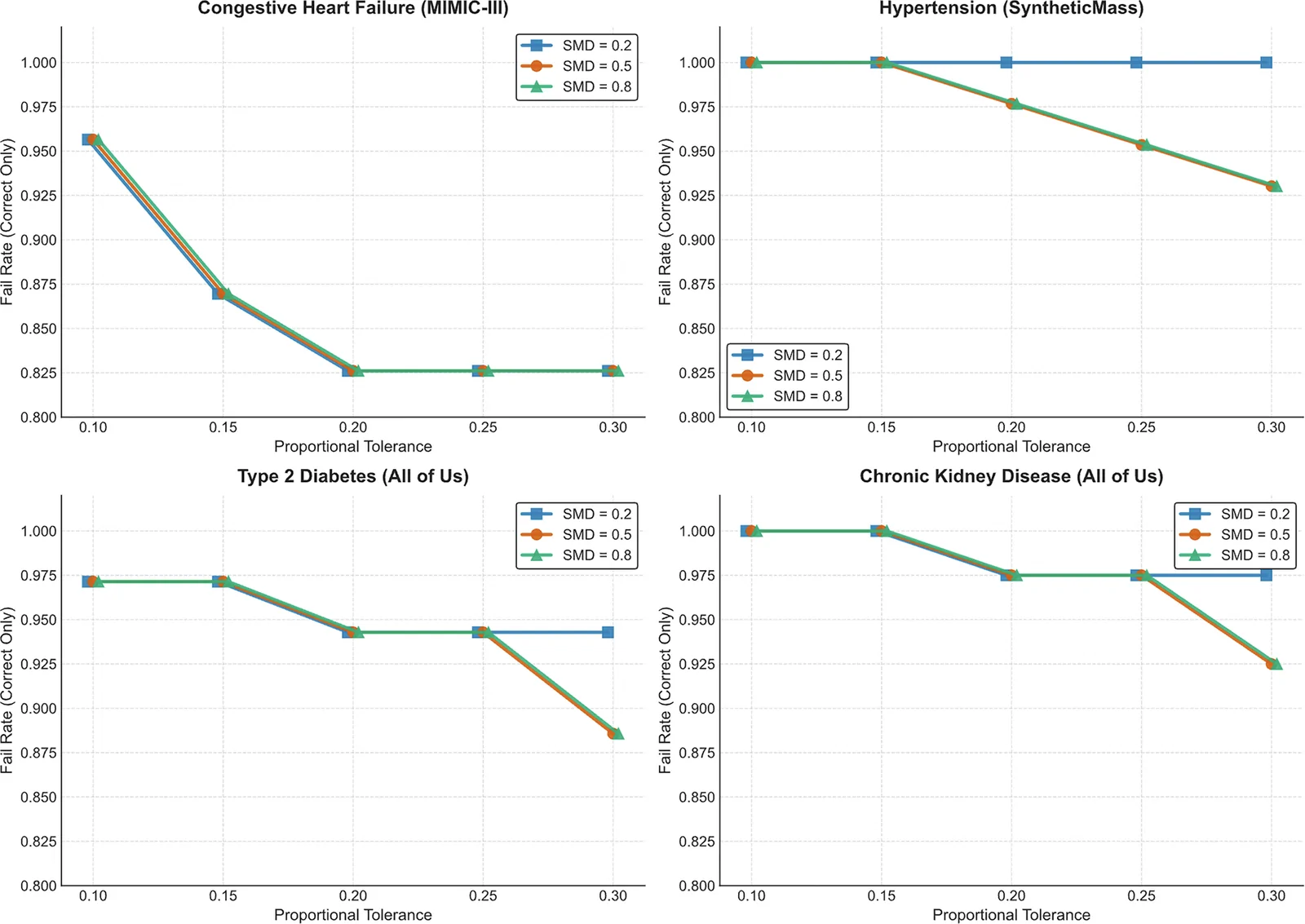
Sensitivity analysis across statistical thresholds for correctly grounded tests. Failure rates across correctly grounded tests are shown for proportional tolerance thresholds of 10% to 30% and SMD cutoffs of 0.2, 0.5, and 0.8. Across cohorts, failure rates varied more with proportional tolerance than with the SMD cutoff, and remained largely stable across SMD values.

We then evaluated the sensitivity of the framework to statistical threshold selection by varying the proportional tolerance threshold from 10% to 30% and the SMD threshold across 0.2, 0.5, and 0.8. As shown in Fig. 3 for correctly grounded tests and in Fig. S3 for all tests, failure rates across all four cohorts were more sensitive to proportional tolerance than to the SMD threshold. By contrast, results remained largely stable across the evaluated SMD cutoffs, with only modest cohort-specific divergence at higher proportional tolerances. Full sensitivity values are provided in Table S11.

## Discussion

In this study, we introduced Medical Data Pecking, a semantic unit testing methodology that adapts software engineering principles to medical data validation. While traditional quality assessment tools (such as OHDSI Achilles) provide extensive libraries of universal checks (e.g., ’Gender=Male cannot have pregnancy codes’), these rules are necessarily context-agnostic. A static rule library cannot capture the complexity of clinical context, such as the expected prevalence of a specific comorbidity in a distinct demographic cohort. Consequently, MDPT functions as a complementary semantic validation layer. By addressing clinical implausibility in syntactically perfect data, the framework can identify discrepancies that may not be detected by traditional integrity checks. Using MDPT we generated 23–43 semantic tests classified as correct and identified 20-43 discrepancies per cohort in data that were syntactically valid under standard checks. This demonstrates the benefit of using adaptive validation rather than a fixed set of rules. Our results suggest that syntactic completeness alone may be insufficient to establish semantic plausibility, and that verification against external evidence may reveal clinically relevant mismatches.

The high rate of discrepancies observed even among accurately grounded tests (Fig. 2C) is consistent with a persistent semantic gap in these datasets. At the same time, these results should not be interpreted as evidence of data failure; rather, they show that MDPT is able to bring these discrepancies to the attention of researchers.

Sensitivity analyses indicated that the main findings were more sensitive to proportional tolerance than to the SMD cutoff. Failure rates remained largely stable across the evaluated SMD values, suggesting that proportional tolerance had the greater influence on evaluation strictness.

Consequently, it is crucial to distinguish between data errors and cohort selection biases. A high failure rate in the MDPT suite does not always imply corrupted data; rather, it quantifies the distance between the specific dataset and the general population baseline as reported in the scientific literature. For example, the high failure rate in MIMIC-III reflects its specific ICU population, which naturally deviates from outpatient epidemiological priors. Similarly, the failures observed in the SyntheticMass dataset may reflect the specific generative methodology of the Synthea engine, where certain complex clinical correlations may not have been modeled, rather than a failure of the data pipeline itself. In this setting, MDPT can help characterize representational discrepancies introduced during synthetic data generation and identify how the resulting study population differs from the general population.

The detection of formatting-induced errors in MIMIC-III highlights how clinically meaningful information can become unavailable to downstream analyses despite remaining syntactically valid in the raw data. Although the underlying code strings are present and pass syntactic checks, the omission of a decimal point can alter the encoded signal enough to make the phenotype effectively undetectable by standard algorithms. This example shows that semantic testing can identify failures that conventional syntactic checks may miss through implausible prevalence estimates. In particular, zero-prevalence failures provide a distinct diagnostic signal that suggests either a technical artifact or a mismatch between the clinical query and the level of abstraction represented in the dataset. By identifying these cases, the framework helps distinguish routine distributional differences from more fundamental incompatibilities between the intended research question and the structure of the available data.

The imperative for this dynamic validation is growing. The White House Office of the National Cyber Director advocated the development of quality metrics to evaluate software modularity, security, and automation^28^. Similarly, the Bipartisan House AI Task Force identified data quality as a critical concern^29^. Alderman et al.^30^ reinforced these concerns through the STANDING Together recommendations, a global initiative to mitigate algorithmic bias in healthcare AI. In the era of Big Data, manual verification is infeasible and errors in the data pipeline propagate unnoticed, undermining the validity of downstream AI models. Our framework provides a scalable and automated approach for addressing this validity gap, offering a mechanism to operationalize these high-level policy and ethical recommendations into code.

A primary challenge in applying LLMs to quality assurance is the risk of hallucination or faulty tests. We addressed this through a tiered evidence grounding protocol reinforced by an automated verification loop. Rather than relying on the LLM’s knowledge, our system functions as a retrieval engine, anchoring every test in verifiable sources. Crucially, the system utilizes a recursive search strategy: after the initial generation, a secondary ’Auditor Agent’ independently searches for and verifies the statistics, allowing the system to self-correct and discard unsupported claims before they become unit tests. Although our evaluation showed that drug prevalence tests remain difficult to ground due to data scarcity, the stronger grounding of disease and demographic tests suggests that RAG-based architectures with independent verification cycles may support automated epidemiological validation.

In this study, we use an auditor agent to reduce major grounding errors. Nevertheless, there is still room for improvement that may be achieved by using more advanced LLMs and by conducting additional iterations of “debate" between agents until they agree on the values reported in the document.

To maintain clinical reliability, the Medical Data Pecking framework separates generative synthesis of tests from their subsequent execution. By fixing the validated test matrix as a versioned asset within the clinical pipeline, the system provides a stable audit trail. This architecture mitigates reproducibility risks associated with evolving external APIs and helps maintain consistency of the audit over time.

As Dahl et al.^31^ argued, testing can be used to show the presence of bugs, but never to show their absence. Our current implementation is limited by its reliance on general population benchmarks, which can obscure deviations in specific subgroups. However, this is largely a limitation of data availability rather than of the methodology itself.

While MDPT focuses on prevalence, the framework’s architecture can be expanded to support deterministic semantic tests, such as temporal coherence and dosage plausibility.

Moreover, the reliance on general population priors introduces a form of ’reverse ecological fallacy’: the assumption that a specialized, highly filtered clinical cohort (such as an ICU population) should mirror broad national averages. This reliance creates an inherent noise floor, where MDPT may flag valid clinical divergence, necessary for the study of specific diseases, as a semantic error.

While temporal mismatch is a systemic challenge, it is the representational discrepancy, specifically formatting-induced errors in clinical coding, that serves as the primary driver for failures in specific phenotypes like CHF. In MIMIC-III, the omission of decimal points in ICD-9 codes prevents automated phenotype algorithms from correctly identifying conditions, creating a semantic gap that likely exceeds the noise introduced by shifting epidemiological priors. However, even with perfect formatting, temporal mismatch remains a critical secondary factor when analyzing historical cohorts. Comparing decade-old clinical signals against contemporary benchmarks introduces an ’era gap’ in diagnostic criteria and prevalence. Thus, while the immediate hurdle is technical (ICD-9 formatting), era-aware grounding is essential for the longitudinal validity of generative semantic testing.

As local health systems digitize their internal baselines, these can replace the general literature as the ground truth. Furthermore, the impact on the validity of subsequent research must be carefully considered; there is a risk of the *implied truth effect* where researchers can interpret the absence of warnings as an indication of validity^32^. Semantic testing is designed to detect known impossibilities, not to certify absolute truth. To mitigate it, we propose that generative auditing must adopt a tri-state reporting protocol where clinical variables are explicitly categorized as Passed, Failed (Semantic Gap), or Inconclusive (No Reference or Out-of-Scope). By treating ’No Reference’ results as a distinct category rather than a default pass, the system ensures that researchers interpret an absence of a flag as a lack of evidence rather than an endorsement of data quality. This transparency may help reduce over-interpretation and support safer integration of Medical Data Pecking into clinical data governance.

It is important to distinguish the methodology (semantic unit testing) from the mechanism (LLMs). While our current implementation relies on GPT-4o and Bing Search, the framework itself is model-agnostic. As foundational models evolve, the ’Auditor Agent’ can be upgraded to specialized biomedical models, but the underlying logic, which is validating data against dynamic external truth, remains the main methodological contribution of this work.

Ultimately, these findings support more routine use of automated data testing pipelines in medical AI. Similarly to modern software development where automated testing pipelines are standard, medical AI may benefit from comparable testing workflows. Furthermore, using such tests continuously on data collected by healthcare institutes can help identify problems at the source.

## Methods

The Medical Data Pecking framework consists of three main components: (i) a taxonomy of semantic unit tests, (ii) automated methods for test synthesis, and (iii) procedures for applying these tests to data. This section describes each component in turn and then presents the datasets used to evaluate MDPT, the reference implementation of the framework.

### Taxonomy of semantic unit tests

To systematically evaluate the extent to which data were validated for research, we propose a hierarchical taxonomy of data validation based on the software engineering principle of “test coverage"^33^. Traditional quality metrics primarily ensure syntactic coverage, verifying that fields adhere to correct formats. While some frameworks extend this to semantic validation, they rely on manually curated rules to detect logical errors. This approach is labor-intensive and not scalable to the complexity of medical data. In contrast, our approach introduces Semantic Data Coverage to validate the alignment of observed data with epidemiological ground truth. Specifically, we distinguish three distinct levels of validation: the syntactic (metadata) level, the distributional semantic level, and the contextual semantic (subpopulation) level.

Syntactic level (Metadata): Tests at this level verify structural integrity, such as field presence, data type correctness, and non-missing values. This serves as a baseline validation to ensure that the data are technically accessible.

Distributional semantic level: Tests at this level validate that population metrics align with theoretical or literature baselines. For example, the distribution of a specific laboratory test across the entire cohort is checked against its expected range. This ensures that the data are statistically plausible in aggregate.

Contextual semantic level (Subpopulation): Tests at this level analyze intra-group patterns, such as comorbidity rates or drug prevalence within specific demographic cohorts. For example, these tests might check the prevalence of hypertension in women compared to men. This level offers greater depth than population checks by detecting context-specific biases or errors that remain invisible to aggregate analysis.

### Core concepts and framework overview

For clarity, we use the following terms throughout the framework:

Semantic Unit Testing: A methodology that adapts software engineering unit testing principles to validate discrete clinical segments of medical data against external scientific knowledge.

Semantic Data Coverage: The extent to which observed data fields are validated for alignment with epidemiological ground truth, moving beyond simple formatting checks.

Semantic Gap: A statistically significant divergence where data maintains Syntactic Integrity (adherence to format and metadata) but lacks Semantic Plausibility (alignment with clinical reality).

**Fig. 4:**
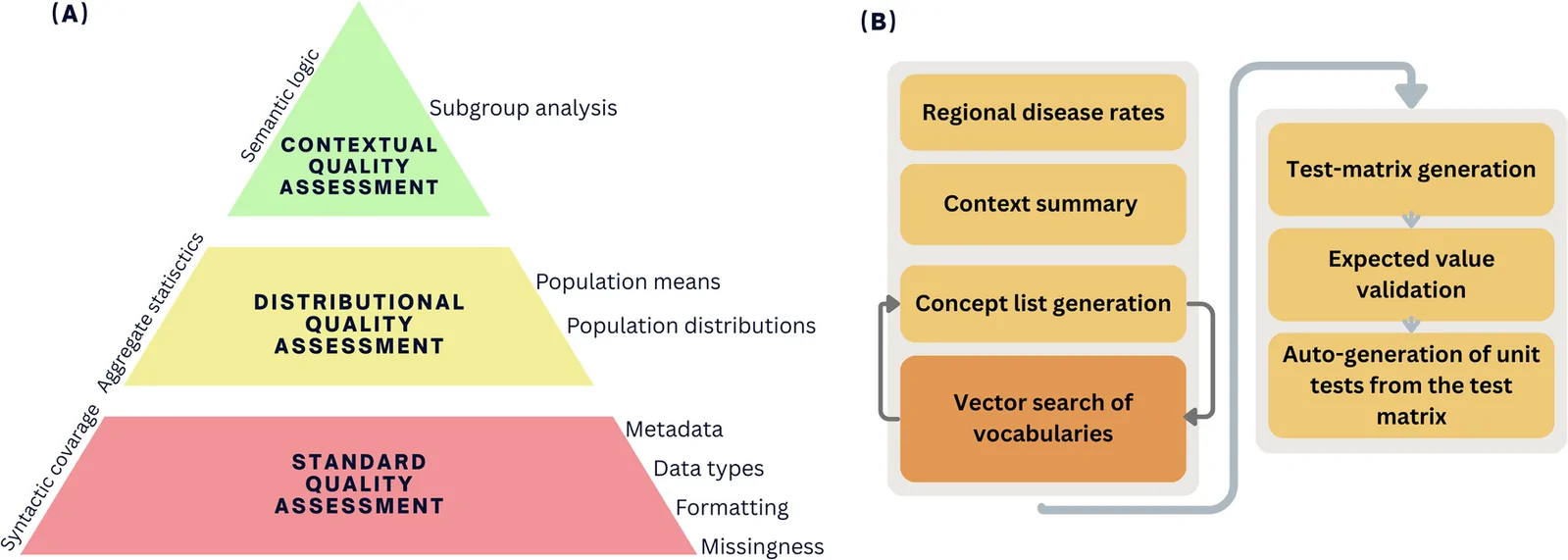
The Semantic Unit Testing Paradigm and MDPT Architecture. **A** The Semantic Gap in data quality. Standard quality assessment tools (red) typically provide only syntactic coverage (metadata and formatting). The proposed framework extends validation to the distributional (yellow) and contextual (green) semantic layers to detect errors in meaning. **B** The generative workflow. The system operates in two stages. In the first stage (left panel), it retrieves epidemiological statistics via web search (Bing) while simultaneously mapping clinical concepts to standard vocabularies via vector search. In the second stage (right panel), it synthesizes these inputs into a test matrix and performs 'Expected Value Validation', a double-check by an Auditor Agent to prevent hallucinations before generating the final unit tests.

A schematic description of the semantic testing depth hierarchy is presented in Fig. 4a. To implement this semantic taxonomy at scale, we developed the MDPT. As illustrated in Fig. 4b, the framework utilizes an LLM to automatically synthesize these tests from the medical literature and run them against the target dataset.

### Automated test synthesis

To support deployment in secure clinical environments, the MDPT framework utilizes a decoupled architecture that separates logic generation from data execution. The system is organized into two functionally isolated modules: the Generation Module and the Test Application Module.

Generation Module: This module operates exclusively on the study specification and the data dictionary, which describes the available database fields, their meanings, formats, and coding. The LLM utilizes this metadata to map clinical intent to the local database schema and generate executable unit tests. No raw, individual-level patient data is ever transmitted to or accessed by this module.

Test Application Module: This module resides within the user’s secure, isolated environment. It receives the generated test suite and executes it directly against the raw patient data. This separation of concerns ensures that protected health information remains within the local security perimeter while utilizing generative models for test construction.

### Contextual parameter extraction and grounding

To address the scalability challenge of creating context-specific unit tests, the MDPT framework employs a Retrieval-Augmented Generation (RAG) architecture. The test generation pipeline operates in two primary stages: Contextual Parameter Extraction and Automated Grounding and Evidence Verification.

Contextual Parameter Extraction: The system accepts a high-level study specification (condition of interest, geographic region, and the medical ontology used) and a data dictionary (Fig. S1). Using an LLM, the system identifies relevant medical concepts using a search engine API and aligns them using OHDSI standardized vocabularies (e.g., OMOP, ICD-9, SNOMED). Rather than performing a traditional source-to-concept mapping, the system utilizes these ontologies as a semantic reference library to define the clinical ground truth for the audit. Full model specifications and prompt templates are detailed in Section S2 in the Supplementary Materials and Tables S2–S6. The specific vocabularies used in MDPT are detailed in Table 1. To ensure accurate concept mapping, standardized vocabularies are pre-embedded using OpenAI’s “text-embedding-3-small" model and stored in a vector database (Chroma). To facilitate concept alignment, a structured metadata-augmented retrieval strategy was employed. For each identified clinical entity, a query string was constructed incorporating the clinical term, domain identifier, and vocabulary source (e.g., ’concept_name: [diagnosis or comorbidity] domain_id: [domain] vocabulary_id: [user provided medical ontology] standard_concept: S’). The system retrieves the top 50 candidate concepts from the Chroma vector database using cosine similarity. From this candidate pool, the LLM performs a secondary semantic verification to select the most appropriate standardized identifiers for the test suite.

**Table 1:** Clinical vocabularies included in the vector database used by MDPT for source code retrieval

Vocabulary ID	Vocabulary Name	Vocabulary Version	Concept Count
ICD10CM	International Classification of Diseases, Tenth Revision, Clinical Modification (NCHS)	ICD10CM FY2025 code descriptions	99421
ICD9CM	International Classification of Diseases, Ninth Revision, Clinical Modification, Volume 1 and 2 (NCHS)	ICD9CM v32 master descriptions	17564
LOINC	Logical Observation Identifiers Names and Codes (Regenstrief Institute)	LOINC 2.77	271441
ICD9Proc	International Classification of Diseases, Ninth Revision, Clinical Modification, Volume 3 (NCHS)	ICD9CM v32 master descriptions	4657
Ethnicity	OMOP Ethnicity	NA	2
Race	Race and Ethnicity Code Set (USBC)	Version 1.0	53
Gender	OMOP Gender	NA	5
ATC	WHO Anatomic Therapeutic Chemical Classification	ATC 2024-07-03	7131
DRG	Diagnosis-related group (CMS)	2011-18-02	1362
RxNorm Extension	OMOP RxNorm Extension	RxNorm Extension 20240701	2146945
PPI	AllOfUs_PPI (Columbia)	Codebook Version 0.4.43 + COVID + MHWB + SDOH + PFH + BHP + EHH	6798
CPT4	Current Procedural Terminology version 4 (AMA)	2024 Release	17455
ICD10	International Classification of Diseases, Tenth Revision (WHO)	2021 Release	16638
SNOMED	Systematic Nomenclature of Medicine - Clinical Terms (IHTSDO)	2024-02-01 SNOMED CT International Edition; 2024-03-01 SNOMED CT US Edition; 2024-04-10 SNOMED CT UK Edition	1084286

The table lists each vocabulary identifier, its full name, its version used, and the total number of indexed concepts.

**Fig. 5:**
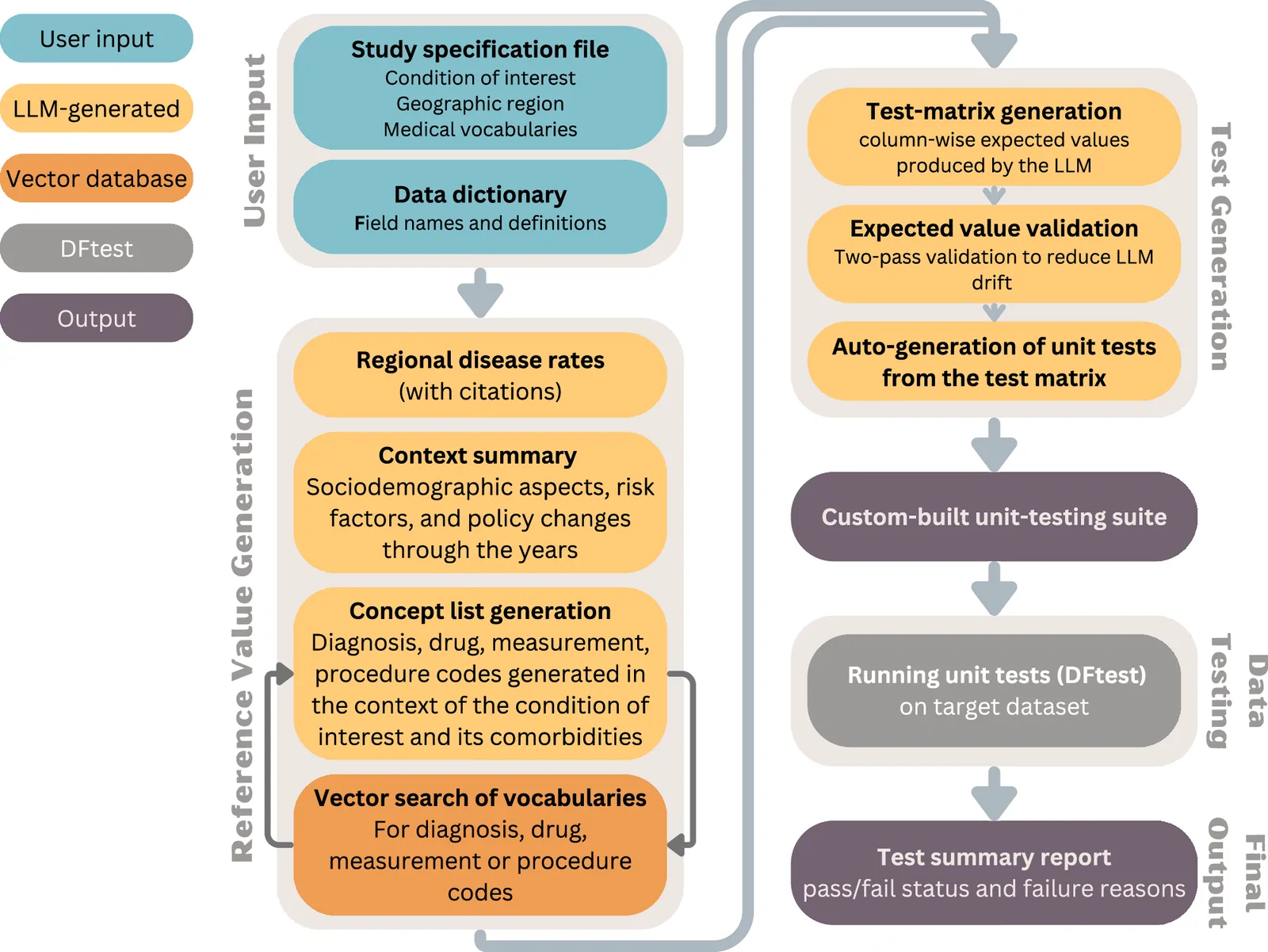
A schematic overview of the MDPT workflow. User-defined study parameters are input into an LLM module that retrieves regional disease rates, contextual epidemiology, and generates lists of diagnosis, drug, measurement, and procedure codes via vector-database search. These reference values are transformed into a test matrix, implemented as unit tests, and validated in a double-pass step to reduce LLM hallucinations. The DFtest framework then executes the suite on the target dataset, yielding binary test outcomes and statistical comparisons between observed data and expected epidemiological distributions.

Automated Grounding and Evidence Verification: To ensure that the tests are anchored in epidemiological reality, we implemented a three-step grounding protocol, which is illustrated in Fig. 5, comprising directed prioritization, test matrix construction, and Auditor Agent verification.

Directed Prioritization: The system retrieves initial reference statistics via the Bing Search API. Through system prompting, the model is explicitly required to prioritize high-authority sources, such as government health agencies (e.g., CDC, NIH) and peer-reviewed literature, over general web results when selecting benchmarks.

Test Matrix Construction: The retrieved epidemiological benchmarks are transformed into a structured test matrix. This matrix defines the preliminary expected value ranges for each semantic test (e.g., “The prevalence of Type 2 Diabetes in US adults is expected to be between 8% and 12%"). This automated synthesis allows the framework to generate dozens of context-aware tests from a single high-level query.

The Auditor Agent (Double-Pass Verification): To mitigate hallucination, we used an approach inspired by Self-Refine^34^, where an “Auditor Agent" performs a new, targeted search to verify the accuracy of the statistics proposed in the matrix. This agent is explicitly required to validate the correctness of the extracted value; it cross-references the matrix entries against newly retrieved evidence and automatically corrects the value if discrepancies are detected. In this implementation, both the Generator and Auditor utilize GPT-4o; however, independence is maintained through information decoupling, where the Auditor initiates an entirely separate search thread and role-specific prompting. This architecture ensures that the verification pass is grounded in a fresh evidentiary context.

### Execution and statistical validation

Unlike software unit tests, which typically require binary pass/fail outcomes, semantic data testing requires statistical tolerance to account for natural population variance and sample size differences. Adopting Software Engineering principles, we define a “test failure" as any objective non-alignment between a clinical assertion and the observed data. This technical definition is agnostic to the root cause; a failure flag may be triggered by technical data inconsistencies (e.g., formatting), scientifically valid cohort-selection effects (e.g., an ICU-specific design), or tool-related discrepancies. We implemented a custom execution framework, DFtest, which applies the generated test matrix to the target dataset. While the synthesis of this matrix involves stochastic API processes, the resulting suite is persisted as a static, version-controlled asset. This decoupling ensures that the execution phase remains unaffected by updates or drift in the underlying commercial models or search algorithms. The framework applies the generated test matrix to the target dataset using three statistical criteria. These values were used as reference calibration settings in the baseline analysis and were not intended to represent universally optimal thresholds across all datasets or variable types:

Distributional Equivalence: For continuous variables (e.g., age at diagnosis), the framework applies Welch’s t-test for unequal variances. A *p*-value less than 0.05 flags a statistically significant deviation from the expected distribution.

Magnitude of Difference: To distinguish between statistical significance and clinical relevance^35^, particularly in large datasets where minor deviations may yield low *p*-values, we calculate the Standardized Mean Difference (SMD). Discrepancies are flagged only when the SMD equals or exceeds 0.2, representing a non-negligible deviation from the expected distribution.

Proportional Tolerance: For categorical prevalence (e.g., comorbidity rates), we define a tolerance margin based on prior EHR validation studies^36^. A test passes if the ratio between the observed and expected proportion falls within the interval of 0.85 to 1.15. Ratios outside this range indicate a deviation greater than 15% from the expected baseline and trigger a failure flag.

### Evaluation datasets

To demonstrate the generalizability of this paradigm, we deployed the framework across three datasets representing distinct data structures and coding systems.

All of Us v7 (controlled tier CDR), from the All of Us research program (https://www.researchallofus.org/). A large-scale U.S. biobank targeting one million participants, with emphasis on historically underrepresented populations (OMOP). Population characteristics are described in Table S8.

MIMIC-III v.1.4^37^, a publicly available de-identified critical care database of over 60,000 admissions to Beth Israel Deaconess Medical Center (ICD-9).

SyntheticMass^38^, a synthetic population dataset simulating the health records of Massachusetts residents (SNOMED-CT).

## Supplementary information


Supplementary Information
Transparent Peer Review file


## Data Availability

The datasets analyzed during the current study are available under different access conditions. All of Us data are not publicly available due to controlled-access restrictions designed to protect participant privacy and are available to eligible researchers through the All of Us Researcher Workbench (https://www.researchallofus.org/). MIMIC-III is available to credentialed users through PhysioNet (https://mimic.mit.edu/). SyntheticMass is publicly available at https://syntheticmass.mitre.org/download.html.

## Code availability

The code used in this study is publicly available on GitHub at https://github.com/TAU-MLwell/mdpt. The implementation was developed in Python, and details of the software environment and dependencies are provided in the repository.

## References

[1] Prokosch, H. U. & Ganslandt, T. Perspectives for medical informatics. Reusing the electronic medical record for clinical research. *Methods Inf. Med.* **48**, 38–44 (2009). 19151882

[2] Seminara, N. et al. Validity of The Health Improvement Network (THIN) for the study of psoriasis **164**. https://www.ncbi.nlm.nih.gov/pmc/articles/PMC3064479/. 10.1111/j.1365-2133.2010.10134.x PMC3064479 21073449

[3] Coorevits, P. et al. Electronic health records: new opportunities for clinical research **274**. https://onlinelibrary.wiley.com/doi/abs/10.1111/joim.12119. 10.1111/joim.12119 23952476

[4] Chen, W. et al. Research data warehouse: using electronic health records to conduct population-based observational studies. *JAMIA Open* **6**, ooad039 (2023). 37359950 10.1093/jamiaopen/ooad039 PMC10284679

[5] Rakers, M. M. et al. Availability of evidence for predictive machine learning algorithms in primary care: a systematic review. *JAMA Netw. Open* **7**. 10.1001/jamanetworkopen.2024.32990. PMC11393722 39264624

[6] Cardozo, G., Tirloni, S. F., Pereira Moro, A. R. & Marques, J. L. B. Use of artificial intelligence in the search for new information through routine laboratory tests: systematic review. *JMIR Bioinform. Biotechnol*. **3**. 10.2196/40473 PMC9828303 36644762

[7] Gjesvik, J., Moshina, N., Lee, C. I., Miglioretti, D. L. & Hofvind, S. Artificial intelligence algorithm for subclinical breast cancer detection. *JAMA Netw. Open* **7**, e2437402 (2024). 39361281 10.1001/jamanetworkopen.2024.37402 PMC11450515

[8] Syed, R. et al. Digital health data quality issues: systematic review. *J. Med. Internet Res.* **25**, e42615 (2023). 37000497 10.2196/42615 PMC10131725

[9] Weiskopf, N. G. & Weng, C. Methods and dimensions of electronic health record data quality assessment: enabling reuse for clinical research. *J. Am. Med. Inform. Assoc.* **20**, 144–51 (2013). 22733976 10.1136/amiajnl-2011-000681 PMC3555312

[10] Sambasivan, N. et al. “Everyone wants to do the model work, not the data work”: Data Cascades in High-Stakes AI. In *Proceedings of the 2021 CHI Conference on Human Factors in Computing Systems*, CHI ’21, 1–15 (Association for Computing Machinery, New York, NY, USA, 2021). 10.1145/3411764.3445518.

[11] Kilkenny, M. F. & Robinson, K. M. Data quality: "garbage in–garbage out”. *Health Inf Manag* 47, 103–105 (2018). 10.1177/1833358318774357 29719995

[12] Kahn, M. G. et al. Transparent reporting of data quality in distributed data networks. *Egems* **3** (2015). 10.13063/2327-9214.1052 PMC4434997 25992385

[13] Mitra, A.*Fundamentals of quality control and improvement* (John Wiley & Sons, 2016).

[14] Ellims, M., Bridges, J. & Ince, D. C. The economics of unit testing. *Empir. Softw. Eng.* **11**, 5–31 (2006).

[15] Shahin, M., Ali Babar, M. & Zhu, L. Continuous integration, delivery and deployment: A systematic review on approaches, tools, challenges and practices. *IEEE Access* **5**, 3909–3943 (2017).

[16] Reimer, A. P. & Madigan, E. A. Veracity in big data: How good is good enough. *Health Informatics J.* **25**, 1290–1298 (2019). 29388495 10.1177/1460458217744369

[17] Lewis, A. E. et al. Electronic health record data quality assessment and tools: a systematic review. *J. Am. Med. Inform. Assoc.* **30**, 1730–1740 (2023). 37390812 10.1093/jamia/ocad120 PMC10531113

[18] Ntinopoulos, V. et al. Large language models for data extraction from unstructured and semi-structured electronic health records: a multiple model performance evaluation. *BMJ Health & Care Informatics* **32** https://informatics.bmj.com/content/32/1/e101139 (2025). 10.1136/bmjhci-2024-101139 PMC11751965 39832824

[19] Alsentzer, E. et al. Zero-shot interpretable phenotyping of postpartum hemorrhage using large language models. *npj Digital Med.* **6**, 1–10 (2023). 10.1038/s41746-023-00957-x PMC10689487 38036723

[20] Yang, J. et al. Enhancing phenotype recognition in clinical notes using large language models: PhenoBCBERT and PhenoGPT. *Patterns* 100887 https://linkinghub.elsevier.com/retrieve/pii/S266638992300288X. (2023). 10.1016/j.patter.2023.100887 PMC10801236 38264716

[21] Tekumalla, R. & Banda, J. M. Towards automated phenotype definition extraction using large language models. *Genomics Inform.* **22**, 21 (2024). 39482749 10.1186/s44342-024-00023-2 PMC11529293

[22] Anand, A., Ostropolets, A., Ryan, P. & Hripcsak, G. Utility of large language models for concept set curation. Tech. Rep. https://www.ohdsi.org/wp-content/uploads/2024/10/56-Anand-Adit_LLMConcpetSetCuration_2024symposium-Adit-Anand.pdf (2025).

[23] Chen, C. & Stadler, T. GenSpectrum Chat: Data Exploration in Public Health Using Large Language Models http://arxiv.org/abs/2305.13821.ArXiv:2305.13821 [cs, q-bio] (2023).

[24] Han, C. et al. Evaluation of GPT-4 for 10-year cardiovascular risk prediction: Insights from the UK Biobank and KOGES data. *iScience* **27**, 109022 (2024). 38357664 10.1016/j.isci.2024.109022 PMC10865411

[25] McDermott, M. B. A., Nestor, B., Argaw, P. & Kohane, I. Event Stream GPT: A Data Pre-processing and Modeling Library for Generative, Pre-trained Transformers over Continuous-time Sequences of Complex Events http://arxiv.org/abs/2306.11547 (2023).

[26] Narayan, A., Chami, I., Orr, L., Arora, S. & Ré, C. Can Foundation Models Wrangle Your Data? http://arxiv.org/abs/2205.09911. ArXiv:2205.09911 [cs] (2022) .

[27] Kathiresan, N. et al. Representation of race and ethnicity in the contemporary US health cohort all of Us Research Program. *JAMA Cardiol* **8**, 859–864 (2023). 37585212 10.1001/jamacardio.2023.2411 PMC10433138

[28] Back to the Building Blocks: A Path Toward Secure and Measurable Software. Tech. Rep., The White House https://www.whitehouse.gov/wp-content/uploads/2024/02/Final-ONCD-Technical-Report.pdf (2024).

[29] Bipartisan House Task Force on Artificial Intelligence. Tech. Rep., U.S. House of Representatives https://www.speaker.gov/wp-content/uploads/2024/12/AI-Task-Force-Report-FINAL.pdf (2024).

[30] Alderman, J. E. et al. Tackling algorithmic bias and promoting transparency in health datasets: the STANDING Together consensus recommendations. *Lancet Digital Health* **7**, e64–e88 (2025). 39701919 10.1016/S2589-7500(24)00224-3 PMC11668905

[31] Dahl, O.-J., Dijkstra, E. W. & Hoare, C. A. R. *Structured programming* (Academic Press Ltd., 1972).

[32] Pennycook, G., Bear, A., Collins, E. T. & Rand, D. G. The implied truth effect: Attaching warnings to a subset of fake news headlines increases perceived accuracy of headlines without warnings. *Manag. Sci.* **66**, 4944–4957 (2020).

[33] Kochhar, P. S., Thung, F. & Lo, D. Code coverage and test suite effectiveness: Empirical study with real bugs in large systems. In *2015 IEEE 22nd International Conference on Software Analysis, Evolution, and Reengineering (SANER)*, 560–564 https://ieeexplore.ieee.org/abstract/document/7081877 (2015).

[34] Madaan, A. et al. Self-refine: Iterative refinement with self-feedback. in Proc. of the 37th International Conference on Neural Information Processing Systems (NIPS, 2023).

[35] Van Rijn, M. H., Bech, A., Bouyer, J. & van den Brand, J. A. Statistical significance versus clinical relevance. *Nephrol. Dial. Transplant*. **32** 10.1093/ndt/gfw385. 28064161

[36] Tatem, K. S. Comparing Prevalence Estimates From Population-Based Surveys to Inform Surveillance Using Electronic Health Records. *Prev. Chronic Dis*. **14**. https://www.cdc.gov/pcd/issues/2017/16_0516.htm. 10.5888/pcd14.160516 PMC5467464 28595032

[37] Johnson, A. E. W. et al. MIMIC-III, a freely accessible critical care database. *Sci. Data* **3**, 160035 (2016). 27219127 10.1038/sdata.2016.35 PMC4878278

[38] Walonoski, J. et al. Synthea: an approach, method, and software mechanism for generating synthetic patients and the synthetic electronic health care record. *J. Am. Med. Inform. Assoc.* **25**, 230-238 (2018). 10.1093/jamia/ocx079 PMC7651916 29025144

