# Supplementary material for: A generative approach for semantic auditing of electronic health records
Source: NPJ Digit Med. 2026 May 30;9:666. doi: 10.1038/s41746-026-02809-w (PMC13522542; doi:10.1038/s41746-026-02809-w)
Supplement: Supplementary file 1 [file 41746_2026_2809_MOESM1_ESM.pdf]

Supplementary material

Contents

S1 Prompt engineering **3**

S2 User provided information and unit test example **3**

List of Tables

S1 **Reference Validity and Grounding Accuracy of MDPT-Generated Tests Across Four Cohorts.** Summary of test quality evaluation for each condition-specific test suite, reporting the percentage of tests with valid references and the distribution of grounding accuracy categories: accurate, inaccurate (within 2 SD), incorrect (greater than 2 SD), qualitative (non-numeric), and irrelevant (no matching reference). Results reflect the variability in literature coverage and grounding success across datasets and clinical contexts. For each table entry other than the overall test count, we expressed the number of tests in the specified category as a proportion of all tests, excluding the metadata test. 5

S2 Test suggestion prompts 6

S3 Test generation prompts 22

S4 Performance metrics of the Auditor Agent across clinical datasets. The table quantifies the automated refinement of generated tests, distinguishing between corrections (realignment with ground truth) and discards (removal of irreconcilable noise). 40

S5 Comprehensive list of tests generated for the Hypertension (SyntheticMass), CHF (MIMIC-III), CKD (AoU), and T2D (AoU) cohorts. 40

S6 Test suggestion validation prompts 48

S7 Test quality validation statistics when tests that evaluate prevalence of drug usage are disregarded. 52

S8 All of Us descriptive statistics. 52

S9 Full results describing MDPT performance on the four datasets, in terms of pass/fail and reference correctness. 53

S10 Failed Tests with Zero Prevalence 54

S11 Sensitivity Analysis Results 57

List of Figures

S1 **An example of user provided study parameters in JSON format**, illustrating the defined cohort, the coding system, and the reference path to the data schema (All of Us). 3

S2 **Example unit test for validating expected data characteristics.** 4

S3 **Failure rates across statistical threshold combinations for all generated tests.** Failure rates for each cohort are shown across proportional tolerance thresholds of 10%, 15%, 20%, 25%, and 30%, with separate curves for SMD cutoffs of 0.2, 0.5, and 0.8, considering all generated tests. 60

```
{
  "population": {
    "diagnosis": "Type 2 diabetes",
    "region" : "US"
    },

  "data": {
    "coding": "OMOP",
    "drug": "OMOP",
    "procedure": "OMOP",
    "lab": "OMOP",
    "data_structure": "All of Us
Registered Tier Dataset v7 CDR Data
Dictionary (R2022Q4R9) - Field (Column)
Generalizations simple minimal.csv"
    }
}
```


Supplementary Figure S1: **An example of user provided study parameters in JSON format**, illustrating the defined cohort, the coding system, and the reference path to the data schema (All of Us).

.

S1 Prompt engineering

Prompts are used to extract information from LLMs. We structured the prompts to be as detailed as possible for the LLM to perform the required task. While it is common to keep context history in API calls, i.e. sending all previous messages and replies, we found that keeping only the system prompt and the required task yields better results, which aligns with the findings of Mirzadeh et al.[1] and Narayan et al.[2]. The used prompts are available in Supplementary Tables S2, S3 and S6.

S2 User provided information and unit test example

Figure S1 illustrates the data provided by the user. This information is used by MDPT to retrieve contextually relevant statistics. Our implementation uses an LLM (GPT-4o, model version 2024-08-06, with a temperature of 0 and seed set to 37, accessed via the Azure OpenAI API service, API version 2024-05-01-preview). MDPT uses these definitions to generate unit tests. An example of such unit test can be found in S2.

```python
def dftest_Fatty_liver_disease_diagnosis_diagnosed(data_df):
    #"American Diabetes Association (ADA) Standards of Medical Care in Diabetes—2023
    (https://diabetesjournals.org/care/article/46/Supplement_1/S1/148923/Standards-of-Medical-Care-in-Diabetes-2023); Younossi et al.,
    'Epidemiology of NAFLD and NASH: Implications for Liver Transplantation,' Hepatology, 2019
    (https://aasldpubs.onlinelibrary.wiley.com/doi/full/10.1002/hep.30257)"
    output_vals = {}
    test_flag = 'ok'
    explanation = 'Among diagnosed: Fatty_liver_disease_diagnosis rate comparison function. Theoretical value is expected to be between
                   55.0 and 70.0.'
    try:
        # Getting a list of patients diagnosed with Type 2 Diabetes
        diagnosed_ids = data_df[data_df['condition_concept_id'].astype(str).isin(['201826', '4099651', '4230254', '45757508', '4193704',
              '40482801', '4151282', '4200875', '3569411', '443732', '4304377', '40485020', '45757474', '4130162'])]['person_id'].unique()
        diagnosed = data_df[data_df['person_id'].isin(diagnosed_ids)]
        codes = ['4159872', '40565506', '37164766', '4058680', '193256'] # Fatty liver diagnosis codes
        data_per = 100 * diagnosed[diagnosed['condition_concept_id'].astype(str).isin(codes)]['person_id'].nunique() /
                   diagnosed['person_id'].nunique() # calculating percentage of diagnosed with fatty liver among T2D patients
        res = (55.0 <= data_per <= 70.0) # checking whether the prevalence falls within the expected range
        output_vals = {'data_per': data_per, 'range_low': 55.0, 'range_high': 70.0}
        if res == False:
            fail_exp = 'Among diagnosed: Fatty_liver_disease_diagnosis evaluation in the examined data is ' + str(data_per)
            explanation += ' ' + fail_exp
    except Exception as e:
        res = False
        test_flag = "error"
        explanation += ' ' + str(e).replace('"', "").replace("'", "")
    finally:
        output_vals['explanation'] = explanation
        output_vals['test_flag'] = test_flag
        dftest_Fatty_liver_disease_diagnosis_diagnosed.explanation = str(output_vals)
        return res
```

Supplementary Figure S2: **Example unit test for validating expected data characteristics.**

	Valid References	Total Tests	Accurate	Inaccurate	Incorrect	Qualitative	Irrelevant
**T2D**	88.1%	60	59.3%	16.9%	20.3%	0%	3.9%
**CKD**	43.9%	67	60.6%	18.2%	12.2%	3.0%	6.1%
**HTN**	93.0%	73	59.7%	6.9%	26.4%	4.2%	2.8%
**CHF**	35.2%	55	42.6%	22.2%	20.4%	1.9%	13.0%

Supplementary Table S1: **Reference Validity and Grounding Accuracy of MDPT-Generated Tests Across Four Cohorts.** Summary of test quality evaluation for each condition-specific test suite, reporting the percentage of tests with valid references and the distribution of grounding accuracy categories: accurate, inaccurate (within 2 SD), incorrect (greater than 2 SD), qualitative (non-numeric), and irrelevant (no matching reference). Results reflect the variability in literature coverage and grounding success across datasets and clinical contexts. For each table entry other than the overall test count, we expressed the number of tests in the specified category as a proportion of all tests, excluding the metadata test.

Test quality validation

To assess test quality, we evaluated reference validity and grounding accuracy. Grounding accuracy was based on source trustworthiness and correctness of the cited reference values. A source was deemed invalid if it led to a nonexistent page or was unrelated to the studied condition. Reference values were categorized as follows:

- **Accurate:** Matches the expected value from a credible source.
- **Inaccurate:** Within 2 standard deviations (SD) of the expected value, or a point estimate where a range is expected.
- **Incorrect:** Deviates by more than 2 SD from the expected value.
- **Qualitative:** Described as a trend or strength of association rather than a numeric value.
- **Irrelevant:** No relevant reference found for the required statistic.

Categories were grouped into ”correct” and ”non-correct” based on the accuracy of the reference values: the ”correct” category includes the ”accurate” group, while ”non-correct” includes all other groups. Test outcomes were considered passed if they matched the reference, and failed otherwise. Test quality results are presented in Table S1.

Supplementary Table S2: Test suggestion prompts

Prior Bing searches

```
searchs = query_bing(f'prevalence of {diagnosis} in {region}')
searchs1 = query_bing(f'incidence of {diagnosis} in {region}')
searchs2 = query_bing(f'mortality rate of {diagnosis} in {region}')
searchs3 = query_bing(f'lifetime risk of {diagnosis} in {region}')
searchs4 = query_bing(f'survival rate of {diagnosis} in {region}')
search1 = query_bing(f'age distribution among {diagnosis} patients in {region}')
search2 = query_bing(f'mean age and standard deviation of {diagnosis} in {region}')
search3 = query_bing(f'Total {region} population size')
search4 = query_bing(f'Number of diagnosed with {diagnosis} in {region}')
```

System prompt

```
You are a public health and medical expert. Answer as concisely as possible.
Answer for the provided diagnosis, region, coding system, and drug classification
system. Make sure the coding in all your answers fits the requirements. The
following questions are about {diagnosis} in {region}. When searching for
references, have them as up-to-date as possible. Search for regional data and
provide references. If regional data is unavailable, reply with global data. If
there is no available data, answer with 'Data is unavailable'. If you are unsure,
answer with 'I am unsure.'
Work step by step.
**When asked for diagnosis codes, provide only the rerquired codes and don't
```

```
randomly assign codes.**
**When asked for any classification codes, provide the required codes without
truncation.**
**Stick to the required coding systems.**
If asked about diagnosis/drug/procedure codes, the fields in the reference data are
’concept_id’ which includes OMOP codes, ’concept_name’ which are the names of the
codes, ’concept_code’ which are the source vocabulary codes, ’vocabulary_id’ is the
name of the source vocabulary.
In OMOP coding, the standard dictionary is SNOMED and RxNorm, and the required field
is concept_id. For exery other coding, the required field is concept_code.
For demographic variables, always include the race, gender and ethnicity
vocabularies.
When asked for csv tables, divide them using a vertical line |. Add the required
title before the csv, exactly as written in the request.
Please avoid other symbols arount the title. Give me your best answer and provide
references. When asked for Python code, CSVs or text, always format them using
triple backticks as follows: ’‘‘‘python’, ’‘‘‘csv’ and ’‘‘‘text’, respectively, and
end with ’‘‘‘’. At the top of each block (Python code, CSV, or text), include the
required title or filename as part of the response. This title or filename must
appear at the start of each block:

‘‘‘python
Filename: example file name
# your python code here
‘‘‘

or

‘‘‘csv
Title: example title
# your csv code here
‘‘‘

If no filename or title was provided in the reqest, please avoid including a
filename in the response.
Please provide references including a **valid** url or a publication for each answer.
Please confirm understanding of these instructions without replying.
```

Prompts - regional statistics

```
What is the prevalence of {diagnosis} in {region}? Provide the percentage of
diagnosed.
Prioritize more recent data, preferably from government sources.
Please use the following information and provide numbers:

{searchs['webPages']['value']}

Numbers are mandatory. Please provide references.
```

```
What is the incidence of {diagnosis} in {region}? Provide the percentage of
diagnosied.
Prioritize more recent data, preferably from government sources.
Please use the following information and provide numbers:

{searchs1['webPages']['value']}

Numbers are mandatory. Please provide references.
```

```
What is the mortality rate of people with {diagnosis} in {region}? Provide the
percentage.
Prioritize more recent data, preferably from government sources.
Please use the following information and provide numbers:

{searchs2['webPages']['value']}

Numbers are mandatory. Please provide references.
```

```
What is the lifetime risk of developing {diagnosis} in {region}? Provide the
percentage.
Prioritize more recent data, preferably from government sources.
Please use the following information and provide numbers:

{searchs3['webPages']['value']}

Numbers are mandatory. Please provide references.
```

```
What is the survival rate of people with {diagnosis} in {region}? Provide the
percentage.
Prioritize more recent data, preferably from government sources.
Please use the following information and provide numbers:

{searchs4['webPages']['value']}

Numbers are mandatory. Please provide references.
```

```
What is the total population size in {region}? Provide the number in millions.
Prioritize more recent data, preferably from government sources.
Please use the following information and provide numbers:

{search3['webPages']['value']}

Numbers are mandatory. Please provide references.
```

```
Please provide statistics for people diagnosed with {diagnosis} in {region}. Include
Prevalence, Incidence, Mortality rate, Lifetime risk, and Survival.
Provide all statistics in percentage. Please use your previous replies as reference.

Arrange them in a csv table with a vertical line as delimiter. Please add
references. Add 'Title: statistics_in_{region}' before the CSV.
Do not add any other symbols (such as dashes or hypens) to the table.
Structure the table as follows: Prevalence | Incidence | Mortality rate |
Lifetime risk | Survival | total population size in millions | reference.
Give me your best answer. Please provide references.
```

```
Please provide mean and standard deviation of {diagnosis} diagnosis age in {region}.
Please use the following information and prioritize more recent data:

{search1['webPages']['value']}

{search2['webPages']['value']}

If there is no information in the provided data, please use your knowledge.
Numbers are mandatory. Please provide references.
```

```
Please provide the number of diagnosed with {diagnosis} in {region}. please write
a full number (e.g instead of '10 million' write 10000000). Use the following
reference:

{search4['webPages']['value']}

If there is no information in the provided data, please provide it based on the
prevalence and total population size.

Numbers are mandatory. Please provide references.
```

```
Please provide the age statistics for {diagnosis} in {region}. Include mean age,
standard deviation, and count.
Use your previous replies as reference. If there is no data in the provided
information, please use your knowledge.

Arrange them in a csv table with a vertical line as delimiter.
Please add references. Add 'Title: age_stats_in_{region}' before the CSV. Do not add
any other symbols (such as dashes or hypens) to the table.
Give me your best answer. Structure the table as follows: mean_age | std | count |
reference. Make sure all columns are filled. Please provide references for the
opercentage values.
```

List of Comorbidities

```
Please provide a list of 15 most prevalent comorbidities among {diagnosis} patients.
The concept names of the comorbidities should appear as they appear in {coding}
dignosis codes.
 provide a comma separated list of the commorbidities separated by '```list' and
 '```'.
```

Comorbidities - vector database and Bing queries

```
omorbidities.append(json_to_df(get_concepts('concept_name: ' + comorbidity + '
domain_id: Condition ' + 'vocabulary_id: ' + coding + ' standard_concept: S',
n_results=100)))
```

```
search = query_bing(f'Prevalence of {comorbidity} among {diagnosis} patients in
{region}')
```

Comorbidities - prompts

```
Please provide a comprehensive and full list of {coding} codes for {comorbidity}.
Provide a full list of codes without truncation.
Use the following table:

{comorbidities[idx]}

Please include all possible options. Start with general {comorbidity} {coding}
codes, preferably standard ones (with "S").
The csv table should include "{coding} codes" and "comorbidity" columns. Write
comorbidity name and a list of all relevant {coding} codes in one row. Separate the
csv using a vertical line as delimiter.
First include all possible {comorbidity} {coding} codes without complications. Work
step by step. Do not generate random codes.Use only the ones provided. Give me your
best answer.
```

```
Please add a "prevalence" column with prevalence of {comorbidity} among {diagnosis}
patients.
Add a reference column with reference to the prevalence value. Prioritize more
recent data, preferably from government sources.
use the following information:

{search['webPages']['value']}

Give me your best answer. Do not change or remove any other column. Separate the csv
using a vertical line as delimiter.
```

```
Please provide a full table of comorbidities, based on the following table:

{comorbidities1}


provide a table with the following columns: comorbidity|{coding} codes|prevalence.
Wrap the csv in '```csv' and '```'.
Use a vertical line as delimiter. Add 'Title: comorbidities_in_{region}' before the
CSV.
```

List of drugs

```
Please provide a list of 15 specific drug names and/or ingredients used to treat
{diagnosis}.
Be as specific as possible. The concept names of the drugs should appear as they
appear in {drug} drug codes.
Provide a comma separated list of the above drugs separated by '```list' and '```'.
```

Drugs - vector database and Bing queries

```
drugs.append(json_to_df(get_concepts('concept_name: ' + drug_name + 'domain_id:
Drug' + 'vocabulary_id: ' + drug + ' standard_concept: S', n_results=140)))
```

```
search = query_bing(f'Percentage of {diagnosis} patients prescribed {drug_name} in
{region}')
```

Drugs - prompts

```
Please provide a comprehensive list of {drug} codes for {drug_name}. Provide a full
list of codes without truncation.
Use the following table:

{drugs[idx]}

Please include all possible options. Start with general {drug_name} {drug} codes,
preferably standard ones (with "S").
The csv table should include "{drug} codes" and "drug" columns. Write a list of all
relevant {drug_name} {drug} codes under "{drug} codes", and drug name under "drug",
in one row. Work step by step. Do not generate random codes, use only the ones
provided.
Arrange them in a csv table with a vertical line as delimiter. Give me your best
answer.
```

```
Please add a "percentage" column with percentage of {diagnosis} patients treated
with the drug and a "reference" column for the percentage information source with
the relevant references. Prioritize more recent data, preferably from government
sources.
use the following information:

{search['webPages']['value']}

Give me your best answer. Do not change or remove any other column.
```

```
Please provide a full table of drugs, based on the following table:

{drugs1}

Please create a csv with the following columns: {drug} codes|drug|percentage|reference.
Wrap the csv in '```csv' and '```'.
Please arange it so all similar codes are aggregated under the '{drug} code' column
without truncation, description of the same drug name under 'drug'.
Use a vertical line as delimiter. Add 'Title: drug_codes_in_{region}' before the CSV.
```

List of labs and procedures

```
Please provide a list of top 25 specific lab tests and procedure names performed on
{diagnosis} patients  in {region}. Be as specific as possible with procedure or lab
test name. Avoid panel names, include individual tests.
The concept names of the procedures and lab tests should appear as they appear in
{procedure} procedure codes and {lab} terminology.
Provide a comma separated list of the lab tests and procedures separated by
'‘‘‘list' and '‘‘‘'.
```

Labs and Procedures - vector database and Bing quieries

```
json_to_df(get_concepts('concept_name: ' + lab + 'domain_id: measurement' +
'vocabulary_id: ' + lab + ' standard_concept: S', n_results=70))
```

```
json_to_df(get_concepts('concept_name: ' + lab + 'domain_id: procedure' +
'vocabulary_id: ' + procedure + ' standard_concept: S', n_results=70))
```

```
search = query_bing(f'Normal range of {lab} in {region}')
```

Labs and procedures - prompts

```
Please provide a comprehensive list of {lab} codes for the following lab test or
procedure:

{Labs[idx]}

Arrange them in a csv table with a vertical line as delimiter. Please include all
possible options.
The csv table should include "procedure codes" and "procedure" columns. Write a list
of all relevant {lab} or {procedure} codes under "procedure codes", and procedure
name under "procedure", in one row.

Work step by step. Do not generate random codes. Provide a full list of codes
without truncation.
Use only the ones provided. Give me your best answer.
```

```
Please add a "normal range" column with normal ranges of results for each of the
lab tests or procedures.
Prioritize more recent data, preferably from government sources. Please use the
following:

{search[’webPages’][’value’]}

Add a "reference" column for the normal range information source with the relevant
references.
Give me your best answer. Do not change or remove any other column.
```

```
Please provide a full table of lab tests and procedures, based on the following
table:

{Labs1}

Please create a csv with the following columns:
{coding} codes|procedure|normal range.
Wrap the csv in ’‘‘‘csv’ and ’‘‘‘’. Use a vertical line as delimiter.
Add ’Title: ’procedure_codes_in_{region}’ before the CSV.
```

General demographic search

```
search1 = query_bing(f’Percentage of {demo} in {region}’)
```

```
search2 = query_bing(f’Percentage of {demo} diagnosed with {diagnosis} in {region}’)
```

Gender or race or ethnicity - vector database and Bing queries

```
search = query_bing(f’Gender identity distribution in {region}’)
```

```
gender_list.append(json_to_df(get_concepts("concept_name: " + demo + 'domain_id:
Gender' + 'standard_concept: S', n_results=50)))

or

race_list.append(json_to_df(get_concepts("concept_name: " + demo + 'domain_id: Race'
+ 'standard_concept: S', n_results=70)))

or

ethnicity_list.append(json_to_df(get_concepts("concept_name: " + demo + 'domain_id:
Ethnicity' + 'standard_concept: S', n_results=50)))
```

List of gender values

```
List all possible {coding} concept names for all possible gender options in
{region}.
Prioritize more recent data, preferably from government sources. Please use the
following as reference as well:

{search['webPages']['value']}

Provide a comma separated list of the concepts separated by '```list' and '```'.
Just write it as single list, do not declare the category title (gender, race,
ethnicity).
```

Gender - prompts

```
Please provide a comprehensive list of {gender or race or ethnicity} {coding} codes
according to:
   {gender_list[idx] or race_list[idx] or ethnicity_list[idx]}.

Arrange them in a csv table with a vertical line as delimiter.
Please include all possible options for {demo}. Drop irrelevant options.
The csv table should include "{coding} code" and "demographic feature" columns.
Each categorical feature - {gender or race or ethnicity} value -  should appear in
a separate row.
For each row, write all the relevant {coding} codes in the "{coding} code" column
without truncation, and the appropriate demographic feature in "demographic feature"
column.
"demographic feature" column should contain the specific gender, race or ethnicity
subtype concept name.
Work step by step. Do not generate random codes. Use only the ones provided.
Give me your best answer.
```

```
Please add "percentage" column with the percentage of {demo} in {region}.
Prioritize more recent data, preferably from government sources. Please use the
following:

{search1['webPages']['value']}

Add a reference in the "reference" column for the percentage information source
with the relevant references.
Give me your best answer. Do not change or remove any other column.
```

```
Please add "diagnosed percentage" column with the percentage of {demo} diagnosed
with {diagnosis} in {region}.
Prioritize more recent data, preferably from government sources. Please use the
following:

{search2['webPages']['value']}

Add a reference in the "reference" column for the diagnosed percentage information
source with the relevant references.
Give me your best answer. Do not change or remove any other column
```

List of race values

```
List all possible {coding} concept names for all possible race options in {region}.
Provide a comma separated list of the concepts separated by ’‘‘‘list’ and ’‘‘‘’.
Just write it as single list, do not declare the category title (gender, race,
ethnicity).
```

List of ethnicity values

```
List all possible {coding} concept names for all possible ethnicity options in
{region}.
Provide a comma separated list of the concepts separated by ’‘‘‘list’ and ’‘‘‘’.
Just write it as single list, do not declare the category title (gender, race,
ethnicity).
```

Stacking all demographics - Bing query

```
search = query_bing(f’gender statistics in {region}’)
```

```
search1 = query_bing(f’race statistics in {region}’)
```

```
search2 = query_bing(f’ethnicity statistics in {region}’)
```

Stacking all demographics - prompt

```
Take the following table:

{demo_list1}
```

```
Arrange it so that all similar codes are aggregated into a single column as a list

under '{coding} code', description of the similar codes under 'demographic feature'.

Add population percentage in {region} under 'Percentage' column based on known
statistics. For the statistics, provide **reliable and valid** references - a url
or a publication.

use the following as reference:

{search['webPages']['value']}

{search1['webPages']['value']}

{search2['webPages']['value']}

Use a vertical line as delimiter.
**Do not** change or remove any other column.
Add 'Title: demography_coding' before the CSV.
Wrap the csv in '```csv' and '```'.
Give me your best answer. Provide references.
```

Stacking all demographics among diagnosed - Bing queries

```
search = query_bing(f'gender statistics among patients with {diagnosis} in {region}')
```

```
search1 = query_bing(f'race statistics among patients with {diagnosis} in {region}')
```

```
search2 = query_bing(f'ethnicity statistics among patients with {diagnosis} in
{region}')
```

Stacking all demographics among diagnosed - prompt

```
Take the following table:

{demo_list1}

Arrange it so that all similar codes are aggregated into a single column as a list
under '{coding} code', description of the similar codes under 'demographic feature'.
Add diagnosed population percentage in {region} under 'Percentage' column based on
known statistics. For the statistics, provide **reliable and valid** references - a
url or a publication.
Make sure the statistics you provide are the percentage of diagnosed patients among
specific demographic groups.

use the following as reference:

{search['webPages']['value']}

{search1['webPages']['value']}

{search2['webPages']['value']}

Use a vertical line as delimiter.
**Do not** change or remove any other column.
Add 'Title: diagnosed_demography_coding' before the CSV.
Wrap the csv in '```csv' and '```'.
Give me your best answer. Provide references.
```

Supplementary Table S3: Test generation prompts

System prompt

```
We will create a table of values to evaluate. Please stick to the provided
information, unless specifically asked to provide additional information which was
not mentioned in the files. Include medical, drug, and procedure classification
coding where relevant. Use {coding} for diagnosis classification codes, {drug} for
drug classification codes, {procedure} for procedures, {lab} for lab measurement
classification codes and {coding} and gender, race and ethnicity classification for
demographic coding.
If unsure, respond with "I am unsure."
Make sure the CSVs do not contain anything that will prevent them from being loaded
into a pandas dataframe.

When asked for CSV tables:
Use | (vertical bar) as the separator.
Enclose the table in ’‘‘‘csv’ and ’‘‘‘’.
Exclude any symbols other than those specified.
Separate the text and table clearly.
Create all tables with the following columns: ’flag’,’column name’,’codes’,’data
type’,’value column name’,’description’,’expected value’,’expected value
max’,’expected value min’,’range low’,’range high’,’comparison type’,’references’

If there are multiple classification codes for a specific concept, provide them as
a list in the ’codes’ column (e.g, if there are multiple codes for a specific
diagnosis, drug, procedure, lab test or a demographic property).
```

Please create a separate row for each subpopulation (e.g. for gender - separate rows for male and for female, as well as for the different race and ethnicity options).

Gender, race and ethnicity categories are in the data dictionary ’description’ column and should be used as the possible code values for the demographic subpopulations.

If there is a single expected value, fill "expected value min" and "expected value max" ’with np.nan’, and fill only "expected value" with the value.
"expected value min" is used when the expected result is greater than the expected value, "expected value max" is used when the expected result is less than the expected value.
If we expect a range of values, fill "range low" and "range high".
’column name’ should contain the name of the column from the provided data dictionary that holds the classifiction codes.
"value column name" should contain the name of the column from the provided data dictionary that holds the recorded value for the test or procedure or measurement, if applicable
If there are multiple medical, drug, lab or procedure classification codes related to a specific concept name, add them as a list in the relevant classification code-related column.
Ensure all tables can be concatenated.

For date-related data, use columns with datetime values, if available.
For each diagnosis ({coding}), drug ({drug}), measurement/lab test ({lab}) and procedure ({procedure}) code, provide **only** the relevant meaning/concept name in the ’description’ column. Double check the meaning and provide references for the statistical values.
Provide your response step by step and offer your best possible answer.
Provide references for all descriptions, code meanings, and for the statistical values.

When asked for CSVs or text, always format them using triple backticks as follows: ’‘‘‘csv’ or ’‘‘‘text’, respectively, and end with ’‘‘‘’. At the top of each block (CSV, or text), include the required title or filename as part of the response. This title or filename must appear at the start of each block:

‘‘‘csv
Title: example title
your csv code here
‘‘‘

If no filename or title was provided in the reqest, please avoid including a filename in the response.
Please confirm understanding of these instructions without replying.

Assistance table for correct column naming

```
You are given a database schema where each row specifies a table name, column name,
and description.
Given the following schema:

{data_struct}

Identify the table name, column name, and description for the field related to
patient's date of birth. Return the result in the following format:

Table: <table_name>
Column: <column_name>
Description: <description>
```

```
Given the following schema:

{data_struct}

Identify the table name, column name, and description for the field that stores
**diagnosis code** or **concept identifier** entries.
Focus on fields that reference standardized vocabularies or classification systems.
Return the result in the following format:

Table: <table_name>
Column: <column_name>
Description: <description>
```

```
Look at the demography and diagnoses using the relevant columns from:

{data_struct}

Identify the table name, column name, and description for the field related to
"diagnosis first date". Return the result in the following format:

Table: <table_name>
Column: <column_name>
Description: <description>
```

```
Look at the demography and diagnoses using the relevant columns from:

{data_struct}

Identify the table name, column name, and description for the field related to
"Person's unique identifier". Return the result in the following format:

Table: <table_name>
Column: <column_name>
Description: <description>
```

```
Based on all your previous answers, create a table with the following columns:
'table_name', 'column_name', 'description', 'generic'.
Populate the table with these specific descriptions: birth date, diagnosis code,
diagnosis first date, and unique person identifier.
Add the following to 'generic' column, according to the proper rows: 'personID',
'birthDate', 'code', 'codeDate'. Inclue all of these.

The 'code' and 'codeDate' columns must refer to the {coding} medical coding system,
excluding any other coding systems that may be present.
'codeDate' should refer to the date and time of the diagnosis.
'birthDate' is the date of birth of the person.
All fields are mandatory. If a field is irrelevant, fill it with np.nan.
Wrap the table in a CSV code block using '```csv' and '```'.
Add the title 'Title: convert_to_datastruct' right before the CSV block.
Use a vertical bar '|' as the column separator, without any extra symbols in the
table.
Include all specified columns in the CSV, and prefer datetime columns where
applicable for date-related data.
Work step by step. Make sure all column_name value's are correct and appear in the
data dictionary. Double check your reply.
Work step by step. Give me your best answer.
```

Comorbidities

```
We are going to check comorbidity prevalence among diagnosed.
Please use the relevant tables and columns the following data dictinary:

{data_struct}

and the {coding} codes provided in the following reference file:

{info}
```

For each comorbidity in the provided reference file, suggest expected values for patients diagnosed with {diagnosis} in {region}.
Please use the description column in the data dictionary to describe the content of the column.
Each column/field name in the provided data dictionary can be evaluated as many times as needed, for as many comorbidities as possible.
Build a csv table with ALL of the following columns:
’flag’, ’column name’, ’codes’, ’data type’,’value column name’,’description’, ’expected value’,’expected value max’, expected value min’, ’range low’, ’range high’,’comparison type’,’references’.

All columns are mandatory. If a field is irrelevant, fill it with np.nan. Do not suggest random population statistics. Only use columns and tables from the provided data dictionary.
Use only codes in the reference file.
The ’flag’ column should indicate the data type and should be filled with one of the following values: ’demography’, ’drug’, ’procedure’, ’lab’ or ’diagnosis’.
’value column name’ should contain the actual name of the column from the provided data dictionary that holds the value.
Before the CSV, include the title: "Title: expected_params_comorbidities". Do not separate it from the CSV code block.
Use the vertical bar | as the CSV separator.
Please include only relevant diagnosis codes in the ’codes’ column.
Surround the CSV table with ‘‘‘csv and ‘‘‘.
In the ’column name’ field, use the corresponding column name from the data dictionary where the comorbidity {coding} code can be found.
For the ’comparison type’, specify whether the comparison is an ’exact value’, ’range’, or ’percentage’. If it is a range, use numeric values only.
Do not suggest random population statistics. Only use columns and tables from the provided data dictionary.
In the ’codes’ column, insert all the relevant {coding} medical codes from the reference file as a list. Each comorbidity should appear in a separate row.
Include the code meaning in the ’description’ column, double check the meaning and provide references for the statistical values.
Keep the ’description’ column short, concise and specific with exact descripton of what is evaluated.
Expected values should be percentage of {diagnosis} patients with the comorbidity.
Use all of the provided information to complete the CSV. Include a full list of drug codes.
Provide as many column/field name evaluations from the given data dictionary as possible.
Make sure the ’description’ column includes the description of the column from the data dictionary, including the meaning of the corresponding {coding} codes.
Provide references for the code meanings and for the statistical values. Work step by step. Give me your best answer.

Drugs

```
We are going to evaluate drug use. Please use the relevant tables in the following
data dictionary:

{data_struct}

and the {coding} codes provided in the following reference file:

{info}

For each column or field name in the provided data dictionary, suggest expected
values for patients diagnosed with {diagnosis} in {region}.
Please use the description column in the data dictionary to describe the content of
the column.
Each field name can be used as many times as needed, for as many drugs as possible.
Build a csv table with ALL of the following columns:
'flag', 'column name', 'codes', 'data type','value column name',
'description','expected value','expected value max', 'expected value min',
'range low', 'range high', 'comparison type','references'.

All columns are mandatory. If a field is irrelevant, fill it with np.nan. Do not
suggest random population statistics. Only use columns and tables from the provided
data dictionary.
Use only codes in the reference file.

The 'flag' column should indicate the data type and should be filled with one of the
following values: 'demography', 'drug', 'procedure', 'lab' or 'diagnosis'.
'value column name' should contain the actual name of the column from the provided
data dictionary that holds the value.
Before the CSV, include the title: "Title: expected_params_drugs". Do not separate it
from the CSV code block.
Use the vertical bar | as the CSV separator.
Surround the CSV table with ```csv and ```.
In the 'column name' field, use the corresponding column/field name from the data
dictionary.
For the 'comparison type', specify whether the comparison is an 'exact value',
'range', or 'percentage'. If it is a range, use numeric values only.
```

```
Do not suggest random population statistics. Only use columns and tables from the
provided data dictionary.
In the 'codes' column, insert all the relevant {drug} drug codes from the reference
file as a list. Each drug should appear in a separate row. Include the code meaning
in the 'description' column, double check the meaning and provide references for the
statistical values.
Keep the 'description' column short, concise and specific with exact descripton of
what is evaluated.
Expected values should be percentage of {diagnosis} patients who use the drug.
Use all of the provided information to complete the CSV.
Provide as many column/field evaluations from the given data dictionary as possible.
For date-related data, prefer columns with datetime values where applicable.
Make sure the 'description' column includes the description of the column from the
data dictionary, including the meaning of the corresponding {coding} codes.
Provide references for the code meanings and for the statistical values. Work step by
step. Give me your best answer.
```

Procedures

```
From the following data dictionary, please extract the table and column names which
contain lab test codes and recorded value:

    {data_struct}

Please include the table name, column name and description for the lab test code and
recorded value.
Structure your answer as follows: 'Lab tests: table name, column name, description'.
```

```
From the following data dictionary, please extract the table and column names with
procedure codes and recorded values in the provided data dictionary.

Please include the table name, column name and description for the lab test code and
recorded value.
If there is no such column, please reply with 'no value column', and include only the
description and code columns.
Structure your answer as follows: 'Procedures: table name, column name, description'.
```

```
Based on your previous answers and the {procedure} procedure codes provided in the
following reference file:

{info}

suggest expected values for the procedure in {region}.

Each column/field name can be used for as many procedures as needed.
Build a csv table with ALL of the following columns:
'flag', 'column name', 'codes', 'data type','value column name','description',
'expected value','expected value max', expected value min', 'range low',
'range high','comparison type','references'.

All columns are mandatory. If a field is irrelevant, fill it with np.nan. Do not
suggest random population statistics. Only use columns and tables from the provided
data dictionary.
Use only codes in the reference file.
The 'flag' column should indicate the data type and should be filled with one of the
following values: 'demography', 'drug', 'procedure', 'lab' or 'diagnosis'.
'value column name' should contain the field name in the provided data dictionary
with the procedure value, if applicable. If it doesn't exist, fill it with 'np.nan'.
Please make sure the correct 'value column name' for procedure value is in the output
csv, if applicable.
Before the CSV, include the title: "Title: expected_params_procedures". Do not
separate it from the CSV code block.
```

Use the vertical bar | as the CSV separator.
Surround the CSV table with ‘‘‘csv and ‘‘‘.
In the ’column name’ field, use the corresponding column name from the data dictionary.
For the ’comparison type’, specify whether the comparison is an ’exact value’, ’range’, or ’percentage’. If it is a range, use numeric values only.
Do not suggest random numbers. Only use columns and tables from the provided data dictionary.
In the ’codes’ column, insert all the relevant {procedure} procedure codes from the reference file as a list.
Each procedure should appear in a separate row.
Please include the code meaning in the ’description’ column, double check the meaning and provide references for the statistical values.
Keep the ’description’ column short, concise and specific with exact descripton of what is evaluated (e.g. ’urinary tract infection icd-9 codes’).
In the values, include only the number, without percentage or units. Expected values should be normal range of values for the evaluated procedure.
Use all of the provided information to complete the CSV.
Provide as many column evaluations from the given data dictionary as possible.
Make sure the ’description’ column includes the description of the column from the data dictionary, including the meaning of the corresponding {coding} codes.
Provide references for the code meanings and for the statistical values. Work step by step. Give me your best answer.

Labs

Based on your previous answers and the {lab} measurement codes provided in the provided reference file.

suggest normal expected values for the lab test in {region}.

Please use the description column in the data dictionary to describe the content of the column.
Each column/field name can be used for as many lab tests as needed.
Build a csv table with ALL of the following columns: ’flag’, ’column name’, ’codes’, ’data type’,’value column name’,’description’,’expected value’,

'expected value max', expected value min','range low', 'range high', 'comparison type','references'.
All columns are mandatory. If a field is irrelevant, fill it with np.nan. Do not suggest random population statistics. Only use columns and tables from the provided data dictionary.
Use only codes in the reference file.
Please use your previous replies.
The 'flag' column should indicate the data type and should be filled with one of the following values: 'demography', 'drug', 'procedure', 'lab' or 'diagnosis'.
'value column name' should contain the field name from the provided data dictionary with the lab test result value, if applicable. If it doesn't exist, fill it with 'np.nan'.
Please make sure the correct field name for 'value column name' is in the output csv.
Before the CSV, include the title: "Title: expected_params_labs". Do not separate it from the CSV code block.
Use the vertical bar | as the CSV separator.
Surround the CSV table with ```csv and ```.
In the 'column name' field, use the corresponding column name from the data dictionary.
For the 'comparison type', specify whether the comparison is an 'exact value', 'range', or 'percentage'. If it is a range, use numeric values only.
Do not suggest random numbers. Only use columns and tables from the provided data dictionary.
In the 'codes' column, insert all the relevant {lab} lab test codes from the reference file as a list.
Each lab test should appear in a separate row. Include the code meaning in the 'description' column, double check the meaning and provide references for the statistical values.
Keep the 'description' column short, concise and specific with exact descripton of what is evaluated.
In the values, include only the number, without percentage or units. Expected values should be normal range of values for the evaluated lab test.
Use all of the provided information to complete the CSV.
Provide as many column evaluations from the given data dictionary as possible.
Make sure the 'description' column includes the description of the column from the data dictionary, including the meaning of the corresponding {coding} codes.
Provide references for the code meanings and for the statistical values. Work step by step. Give me your best answer.

Creation of lab and procedure test suggestion CSVs

```
Please provide a single and final csv file for labs based on your previous answer.
Include the required title and wrap it between '```csv' and '```'.
```

```
Please provide a single and final csv file for procedures based on your previous
answers. **Make sure there are no duplications with the labs csv**.
Include the required title and wrap it between '```csv' and '```'.
```

General population demographics

```
Look at the diagnosis-related table and columns in the following data dictionary:

{data_struct}

In the description, what are the available gender classification codes, according to
the description column? Please provide the codes and their meanings.
```

```
Look at the diagnosis-related table and columns in the provided data dictionary.
In the description, what are the available ethnicity classification codes, according
to the description column? Please provide the codes and their meanings.
```

```
Look at the diagnosis-related table and columns in the provided data dictionary.
In the description, what are the available race classification codes, according to
the description column? Please provide the codes and their meanings.
```

```
According to the data dictionary, your previous answers and the following reference
file:

{info}

Please use the coding codes and categorizations provided in the reference file and
```

the data dictionary to suggest expected values for the general population
demographics in {region}.

For each column/field name, suggest expected percentage values for the **general
population** in {region}, according to the provided data.
Please use the description column in the data dictionary to describe the content of
the column.
You can reuse each data field as many times as necessary to represent various
population characteristics and subpopulations.

Create a CSV table with the following columns:
’flag’,’column name’,’codes’,’data type’,’value column name’,’description’,
’expected value’,’expected value max’,’expected value min’,’range low’,
’range high’,’comparison type’,’references’

Follow these guidelines:
All columns are mandatory. If a field is irrelevant, fill it with np.nan.
Each demographic subpopulation should appear in a separate row (e.g a row for female,
male, white, etc).
The possible values for gender, race and ethnicity are stated in the ’description’
column in the provided data dictionary for the relevant data field. Please use them
as well.
Please prefer the codes provided in the data dictionary and only then the codes in
the reference file. You may add all options in the provided data for each
subpopulation.
The ’flag’ column should indicate the data type and should be filled with one of the
following values: ’demography’, ’drug’, ’procedure’, or ’diagnosis’.
’value column name’ should contain the actual name of the column from the provided
data dictionary that holds the value.
Before the CSV, include the title: "Title: expected_params_genpop_demo". Do not
separate it from the CSV code block.
Use the vertical bar | as the CSV separator.
Surround the CSV table with ‘‘‘csv and ‘‘‘.
In the ’column name’ field, use the corresponding column/field name from the data
dictionary.
For the ’comparison type’, specify whether the comparison is an ’exact value’,
’range’, or ’percentage’. If it is a range, use numeric values only.
If the provided expected value is a range, fill "range low" and "range high" and set
’comparison type’ to be a ’range’.

Do not suggest random population statistics. Only use columns and tables from the provided data dictionary.
In the ’codes’ column, for each gender, race and ethnicity subpopulation insert relevant classification codes **from the reference file, your previous answers and the data dictionary** as a list.
Add the relevant gender codes, race codes and ethnicity codes from your previous answers to the appropriate list under the ’codes’ column. The codes that don’t have their own row should appear in ’other’ race, gender or ethnicity row.
Each demographic subpopulation should appear in a separate row (e.g a row for male, a row for female, a row for white, a row for asian, etc.). Include the code meaning in the ’description’ column, double check the meaning and provide references for the statistical values. Keep the ’description’ column short, concise and specific with exact descripton of what is evaluated.
Use all of the provided information to complete the CSV.
Provide as many column evaluations from the given data dictionary as possible.
For date-related data, prefer columns with datetime values where applicable.
Make sure the ’description’ column includes the description of the column from the data dictionary, including the meaning of the corresponding {coding} codes.
If there are any additional demographic {coding} codes that might apply to {region}, please **add** them to the existing list - to check for additional codes you may use your intrinsic knowledge.
Provide references for the code meanings and for the statistical values.
double check your reply.
Please make sure there is a separate row for each subpopulation (e.g. for gender - separate rows for male and for female, as well as for the different race and ethnicity options).
The codes should correspond. Provide your best answer.

Please provide a single and final csv file for the general population demographics based on your previous answers. Include all gender, race, ethnicity and any other categorical fields.
Make sure all relevant gender, race and ethnicity classification codes are included in the ’codes’ column.
Include the required title and wrap it between ’‘‘‘csv’ and ’‘‘‘’.

Please make sure that the previous csv file contains a separate row for each demographic subgroup (e.g a row for female, male, white, etc).
If it does not, please recreate it so it follows the required gudelines.

Diagnosed population demographics

```
According to the data dictionary, your previous answers and the provided reference
file.

Please use the coding codes and categorizations provided in the reference file and
the data dictionary to suggest expected values for the {diagnosis} diagnosed
population demographics in {region}.
For each column/field name, suggest expected demographic statistics values for the
population diagnosed with {diagnosis} in {region}.
Please use the description column in the data dictionary to describe the content of
the column.
Use each column as needed for as many population characteristics as possible. Build a
CSV table with all the following columns:
'flag','column name','codes','data type','value column name','description',
'expected value','expected value max','expected value min','range low',
'range high','comparison type','references'

Follow these guidelines:
All columns are mandatory. If a field is irrelevant, fill it with np.nan.
Each demographic subpopulation should appear in a separate row (e.g a row for female,
male, white, etc).
The possible values for gender, race and ethnicity are stated in the 'description'
column in the provided data dictionary for the relevant data field. Please use them
as well.
Please prefer the codes provided in the data dictionary and only then the codes in
the reference file. You may add all options in the provided data for each
subpopulation.
The 'flag' column should indicate the data type and should be filled with one of the
following values: 'demography', 'drug', 'procedure', 'lab' or 'diagnosis'.
'value column name' should contain the actual name of the column from the provided
data dictionary that holds the value.
```

Before the CSV, include the title: "Title: expected_params_diagnosed_genpop". Do not separate it from the CSV code block.
Use the vertical bar | as the CSV separator.
Surround the CSV table with ‘‘‘csv and ‘‘‘.
In the ’column name’ field, use the corresponding column name from the data dictionary.
For the ’comparison type’, specify whether the comparison is an ’exact value’, ’range’, or ’percentage’. If it is a range, use numeric values only.
If the provided expected value is a range, fill "range low" and "range high" and set ’comparison type’ to be a ’range’.
Do not suggest random population statistics. Only use columns and tables from the provided data dictionary.
In the ’codes’ column, for each gender, race and ethnicity subpopulation insert relevant classification codes **from the reference file, your previous answers and the data dictionary** as a list.
Add the relevant gender codes, race codes and ethnicity codes from your previous answers to the appropriate list under the ’codes’ column. The codes that don’t have their own row should appear in ’other’ race, gender or ethnicity row.

Each demographic subpopulation should appear separately (e.g a row for male, a row for female, a row for white, a row for asian, etc.). Include the code meaning in the ’description’ column, double check the meaning and provide references for the statistical values. Keep the ’description’ column short, concise and specific with exact descripton of what is evaluated - state the exact gender or race or etnicity evaluated.
In the ’expected value’, ’expected value min’, ’expected value max’ columns, provide the percentage of **diagnosed population** with the specific demographic characteristic.
Use all of the provided information to complete the CSV.
Provide as many column evaluations from the given data dictionary as possible.
For date-related data, prefer columns with datetime values where applicable.
Make sure the ’description’ column includes the description of the column from the data dictionary, including the meaning of the corresponding {coding} codes.
If there are any additional demographic {coding} codes that might apply to {region}, please **add** them to the existing list - to check for additional codes you may use your intrinsic knowledge.
Provide references for the code meanings and the values. Alwaus double check your reply. Always work step by step.
Please create a separate row for each subpopulation (e.g. for gender - separate rows for male and for female, as well as for the different race and ethnicity options).

Please provide a single and final csv file for the **diagnosed population demographics** based on your previous answers. Include all gender, race, ethnicity and any other categorical fields.
Make sure all relevant gender, race and ethnicity classification codes are included in the ’codes’ column.
Include the required title and wrap it between ’‘‘‘csv’ and ’‘‘‘’.

Please make sure that the previous csv file contains a separate row for each demographic subgroup (e.g a row for female, male, white, etc).
If it does not, please recreate it so it follows the required gudelines.

Demographic code and test validation

Please use the following data dictionary to answer the following questions: \n\n + {data_struct}

This is a csv containing proposed evaluations: \n\n {messages4[-1][’content’]}

Are there additional relevant codes in one of the rows in the provided table above, that correspond with the value under ’description’ column in each row?
Please return the same table with the same title, with **additional** codes in each row, if applicable. Provide references.
Do not change anything other than what was requested.
Please work step by step and provide your best answer

Disease statistics validation

Are there additional relevant codes in one of the rows in the provided table above, that correspond with the value under ’description’ column in each row?
Please return the same table with the same title, with additional codes in each row, if applicable. Provide references. Ignore this request for the ’convert_to_datastruct’, ’expected_params_procedures’, ’expected_params_drugs’, and ’expected_params_comorbidities’ tables.
Please work step by step and provide your best answer.

```
Make sure the proposed column names for evaluation correspond with the ones in the
provided data dictionary and with the requirements in the system message.

If it doesn't correspond to what was reuired, please change the evaluated column
names according to what was required.
Please work step by step and provide your best answer.
```

```
Make sure the evaluated values correspond with the ones in the provided supplementary
data, data dictionary and the requirements in the first message.
If it doesn't correspond to what was reuired, please change the values according to
what is evaluated.
Please work step by step, provide references and provide your best answer.
```

Supplementary Table S4: Performance metrics of the Auditor Agent across clinical datasets. The table quantifies the automated refinement of generated tests, distinguishing between corrections (realignment with ground truth) and discards (removal of irreconcilable noise).

Dataset	Original	Kept	Corrected	Discarded	Added	Corr. rate (%)	Disc. rate (%)	Kept rate (%)
CHF	60	20	33	6	0	55.00	10.00	33.30
CKD	96	45	43	8	0	44.80	8.30	46.90
HTN	78	26	45	7	1	57.70	9.00	33.30
T2D	94	45	37	2	0	39.40	2.10	47.90

Supplementary Table S5: Comprehensive list of tests generated for the Hypertension (SyntheticMass), CHF (MIMIC-III), CKD (AoU), and T2D (AoU) cohorts.

Cohort	Test Name
Hypertension - SyntheticMass	check data types
Hypertension - SyntheticMass	check incidence
Hypertension - SyntheticMass	check prevalence
Hypertension - SyntheticMass	calculate age distribution
Hypertension - SyntheticMass	Diabetes mellitus diagnosis code among diagnosed
Hypertension - SyntheticMass	Hyperlipidemia diagnosis code among diagnosed
Hypertension - SyntheticMass	Coronary artery disease diagnosis code among diagnosed
Hypertension - SyntheticMass	Obesity diagnosis code among diagnosed
Hypertension - SyntheticMass	Heart failure diagnosis code among diagnosed
Hypertension - SyntheticMass	Atrial fibrillation and related conditions diagnosis code among diagnosed
Hypertension - SyntheticMass	Stroke and related conditions diagnosis code among diagnosed
Hypertension - SyntheticMass	Peripheral vascular disease diagnosis code among diagnosed
Hypertension - SyntheticMass	Chronic obstructive pulmonary disease diagnosis code among diagnosed
Hypertension - SyntheticMass	Sleep apnea and related conditions diagnosis code among diagnosed
Hypertension - SyntheticMass	Depression diagnosis code among diagnosed
Hypertension - SyntheticMass	Osteoarthritis diagnosis code among diagnosed
Hypertension - SyntheticMass	Anemia diagnosis code among diagnosed
Hypertension - SyntheticMass	Male gender
Hypertension - SyntheticMass	Female gender
Hypertension - SyntheticMass	White race

Cohort	Test Name
Hypertension - SyntheticMass	Black or African American race
Hypertension - SyntheticMass	Asian race
Hypertension - SyntheticMass	American Indian or Alaska Native race
Hypertension - SyntheticMass	Native Hawaiian or Other Pacific Islander race
Hypertension - SyntheticMass	Other race
Hypertension - SyntheticMass	Hispanic or Latino ethnicity
Hypertension - SyntheticMass	Not Hispanic or Latino ethnicity
Hypertension - SyntheticMass	Lisinopril
Hypertension - SyntheticMass	Amlodipine
Hypertension - SyntheticMass	Losartan
Hypertension - SyntheticMass	Hydrochlorothiazide
Hypertension - SyntheticMass	Metoprolol
Hypertension - SyntheticMass	Atenolol
Hypertension - SyntheticMass	Valsartan
Hypertension - SyntheticMass	Enalapril
Hypertension - SyntheticMass	Carvedilol
Hypertension - SyntheticMass	Furosemide
Hypertension - SyntheticMass	Clonidine
Hypertension - SyntheticMass	Male gender diagnosed with hypertension
Hypertension - SyntheticMass	Female gender diagnosed with hypertension
Hypertension - SyntheticMass	White race diagnosed with hypertension
Hypertension - SyntheticMass	Black or African American race diagnosed with hypertension
Hypertension - SyntheticMass	Asian race diagnosed with hypertension
Hypertension - SyntheticMass	American Indian or Alaska Native race diagnosed with hypertension
Hypertension - SyntheticMass	Native Hawaiian or Other Pacific Islander race diagnosed with hypertension
Hypertension - SyntheticMass	Hispanic or Latino ethnicity diagnosed with hypertension
Hypertension - SyntheticMass	Not Hispanic or Latino ethnicity diagnosed with hypertension
Hypertension - SyntheticMass	Blood pressure measurement
Hypertension - SyntheticMass	Lipid panel measurement
Hypertension - SyntheticMass	Serum glucose level
Hypertension - SyntheticMass	Serum cholesterol level
Hypertension - SyntheticMass	Serum HDL cholesterol level
Hypertension - SyntheticMass	Serum LDL cholesterol level
Hypertension - SyntheticMass	Serum triglycerides level
Hypertension - SyntheticMass	Serum potassium level
Hypertension - SyntheticMass	Serum creatinine level
Hypertension - SyntheticMass	Serum sodium level

Cohort	Test Name
Hypertension - SyntheticMass	Serum calcium level
Hypertension - SyntheticMass	Serum magnesium level
Hypertension - SyntheticMass	Serum chloride level
Hypertension - SyntheticMass	Serum bicarbonate level
Hypertension - SyntheticMass	Serum urea nitrogen level
Hypertension - SyntheticMass	Serum uric acid level
Hypertension - SyntheticMass	Serum total protein level
Hypertension - SyntheticMass	Serum albumin level
Hypertension - SyntheticMass	Serum globulin level
Hypertension - SyntheticMass	Serum bilirubin level
Hypertension - SyntheticMass	Serum alkaline phosphatase level
Hypertension - SyntheticMass	Serum aspartate aminotransferase level
Hypertension - SyntheticMass	Serum alanine aminotransferase level
Hypertension - SyntheticMass	Serum gamma glutamyl transferase level
Hypertension - SyntheticMass	Serum lactate dehydrogenase level
Hypertension - SyntheticMass	Serum C reactive protein level
CHF - MIMIC-III	check data types
CHF - MIMIC-III	check incidence
CHF - MIMIC-III	check prevalence
CHF - MIMIC-III	calculate age distribution
CHF - MIMIC-III	Hypertension diagnosis codes among diagnosed
CHF - MIMIC-III	Diabetes Mellitus diagnosis codes among diagnosed
CHF - MIMIC-III	Chronic Obstructive Pulmonary Disease diagnosis codes among diagnosed
CHF - MIMIC-III	Atrial Fibrillation diagnosis codes among diagnosed
CHF - MIMIC-III	Coronary Artery Disease diagnosis codes among diagnosed
CHF - MIMIC-III	Anemia diagnosis codes among diagnosed
CHF - MIMIC-III	Renal Failure diagnosis codes among diagnosed
CHF - MIMIC-III	Obesity diagnosis codes among diagnosed
CHF - MIMIC-III	Hyperlipidemia diagnosis codes among diagnosed
CHF - MIMIC-III	Depression diagnosis codes among diagnosed
CHF - MIMIC-III	Peripheral Vascular Disease diagnosis codes among diagnosed
CHF - MIMIC-III	Sleep Apnea diagnosis codes among diagnosed
CHF - MIMIC-III	Hypothyroidism diagnosis codes among diagnosed
CHF - MIMIC-III	Stroke diagnosis codes among diagnosed
CHF - MIMIC-III	Liver Disease diagnosis codes among diagnosed
CHF - MIMIC-III	Male gender
CHF - MIMIC-III	Female gender
CHF - MIMIC-III	White race

Cohort	Test Name
CHF - MIMIC-III	Black or African American race
CHF - MIMIC-III	Asian race
CHF - MIMIC-III	Hispanic Latino ethnicity
CHF - MIMIC-III	Other ethnicity
CHF - MIMIC-III	Lisinopril
CHF - MIMIC-III	Carvedilol
CHF - MIMIC-III	Furosemide
CHF - MIMIC-III	Spironolactone
CHF - MIMIC-III	Digoxin
CHF - MIMIC-III	Valsartan
CHF - MIMIC-III	Sacubitril Valsartan
CHF - MIMIC-III	Bisoprolol
CHF - MIMIC-III	Ivabradine
CHF - MIMIC-III	Eplerenone
CHF - MIMIC-III	Torsemide
CHF - MIMIC-III	Male gender diagnosed with CHF
CHF - MIMIC-III	Black or African American race diagnosed with CHF
CHF - MIMIC-III	Asian race diagnosed with CHF
CHF - MIMIC-III	Hispanic Latino ethnicity diagnosed with CHF
CHF - MIMIC-III	Other ethnicity diagnosed with CHF
CHF - MIMIC-III	Radiologic examination chest single view
CHF - MIMIC-III	Transferase aspartate amino AST SGOT
CHF - MIMIC-III	Alanine aminotransferase ALT measurement
CHF - MIMIC-III	B type Natriuretic Peptide BNP measurement
CHF - MIMIC-III	Troponin I measurement
CHF - MIMIC-III	Troponin T measurement
CHF - MIMIC-III	Serum Creatinine measurement
CHF - MIMIC-III	Blood Urea Nitrogen BUN measurement
CHF - MIMIC-III	Serum Electrolytes measurement
CHF - MIMIC-III	Serum Magnesium measurement
CHF - MIMIC-III	Serum Calcium measurement
CHF - MIMIC-III	Serum Phosphate measurement
CHF - MIMIC-III	Serum Glucose measurement
CKD - All of Us	check data types
CKD - All of Us	check incidence
CKD - All of Us	check prevalence
CKD - All of Us	calculate age distribution
CKD - All of Us	Hypertension diagnosis codes among diagnosed
CKD - All of Us	Heart failure diagnosis codes among diagnosed

Cohort	Test Name
CKD - All of Us	Diabetes Mellitus without complication diagnosis codes among diagnosed
CKD - All of Us	Diabetes Mellitus with complication diagnosis codes among diagnosed
CKD - All of Us	Hyperlipidemia diagnosis codes among diagnosed
CKD - All of Us	Coronary Artery Disease diagnosis codes among diagnosed
CKD - All of Us	Peripheral Vascular Disease diagnosis codes among diagnosed
CKD - All of Us	Depression diagnosis codes among diagnosed
CKD - All of Us	Osteoporosis diagnosis codes among diagnosed
CKD - All of Us	Osteopenia diagnosis codes among diagnosed
CKD - All of Us	Gout diagnosis codes among diagnosed
CKD - All of Us	Sleep Apnea diagnosis codes among diagnosed
CKD - All of Us	Hyperparathyroidism diagnosis codes among diagnosed
CKD - All of Us	Liver Disease diagnosis codes among diagnosed
CKD - All of Us	Female gender
CKD - All of Us	Male gender
CKD - All of Us	White race
CKD - All of Us	Black or African American race
CKD - All of Us	Asian race
CKD - All of Us	Native Hawaiian or Other Pacific Islander race
CKD - All of Us	American Indian or Alaska Native race
CKD - All of Us	Not Hispanic or Latino ethnicity
CKD - All of Us	Hispanic or Latino ethnicity
CKD - All of Us	Lisinopril
CKD - All of Us	Amlodipine
CKD - All of Us	Metoprolol
CKD - All of Us	Furosemide
CKD - All of Us	Erythropoietin
CKD - All of Us	Calcitriol
CKD - All of Us	Cinacalcet
CKD - All of Us	Female gender diagnosed with CKD
CKD - All of Us	White race diagnosed with CKD
CKD - All of Us	Black or African American race diagnosed with CKD
CKD - All of Us	Asian race diagnosed with CKD
CKD - All of Us	Native Hawaiian or Other Pacific Islander race diagnosed with CKD
CKD - All of Us	American Indian or Alaska Native race diagnosed with CKD
CKD - All of Us	Not Hispanic or Latino ethnicity diagnosed with CKD
CKD - All of Us	Hispanic or Latino ethnicity diagnosed with CKD

Cohort	Test Name
CKD - All of Us	Serum Creatinine Measurement
CKD - All of Us	Blood Urea Nitrogen Measurement
CKD - All of Us	Serum Potassium Measurement
CKD - All of Us	Serum Sodium Measurement
CKD - All of Us	Serum Calcium Measurement
CKD - All of Us	Serum Phosphorus Measurement
CKD - All of Us	Serum Albumin Measurement
CKD - All of Us	Hemoglobin Measurement
CKD - All of Us	Hematocrit Measurement
CKD - All of Us	Glomerular Filtration Rate Measurement
CKD - All of Us	Urine Protein Measurement
CKD - All of Us	Urine Albumin Measurement
CKD - All of Us	Urine Creatinine Measurement
CKD - All of Us	Parathyroid Hormone Measurement
CKD - All of Us	Serum Bicarbonate Measurement
CKD - All of Us	Serum Chloride Measurement
CKD - All of Us	Serum Magnesium Measurement
CKD - All of Us	Serum Uric Acid Measurement
CKD - All of Us	Serum Total Protein Measurement
CKD - All of Us	Serum Alkaline Phosphatase Measurement
CKD - All of Us	Serum Aspartate Aminotransferase Measurement
CKD - All of Us	Serum Alanine Aminotransferase Measurement
CKD - All of Us	Serum Bilirubin Measurement
CKD - All of Us	Serum Ferritin Measurement
CKD - All of Us	Serum Transferrin Measurement
T2D - All of Us	check data types
T2D - All of Us	check incidence
T2D - All of Us	check prevalence
T2D - All of Us	calculate age distribution
T2D - All of Us	Hypertension diagnosis among diagnosed
T2D - All of Us	Hyperlipidemia diagnosis among diagnosed
T2D - All of Us	Coronary artery disease diagnosis among diagnosed
T2D - All of Us	Chronic kidney disease diagnosis among diagnosed
T2D - All of Us	Peripheral vascular disease diagnosis among diagnosed
T2D - All of Us	Retinopathy diagnosis among diagnosed
T2D - All of Us	Stroke diagnosis among diagnosed
T2D - All of Us	Depression diagnosis among diagnosed
T2D - All of Us	Osteoarthritis diagnosis among diagnosed
T2D - All of Us	Sleep apnea diagnosis among diagnosed

Cohort	Test Name
T2D - All of Us	Fatty liver disease diagnosis among diagnosed
T2D - All of Us	Anemia diagnosis among diagnosed
T2D - All of Us	Female gender
T2D - All of Us	Male gender
T2D - All of Us	White race
T2D - All of Us	Black or African American race
T2D - All of Us	Asian race
T2D - All of Us	Native Hawaiian or Other Pacific Islander race
T2D - All of Us	American Indian or Alaska Native race
T2D - All of Us	Other race
T2D - All of Us	Not Hispanic or Latino ethnicity
T2D - All of Us	Hispanic or Latino ethnicity
T2D - All of Us	Metformin
T2D - All of Us	Sitagliptin
T2D - All of Us	Saxagliptin
T2D - All of Us	Male gender diagnosed with T2D
T2D - All of Us	White race diagnosed with T2D
T2D - All of Us	Black or African American race diagnosed with T2D
T2D - All of Us	Asian race diagnosed with T2D
T2D - All of Us	Native Hawaiian or Other Pacific Islander race diagnosed with T2D
T2D - All of Us	Not Hispanic or Latino ethnicity diagnosed with T2D
T2D - All of Us	Hispanic or Latino ethnicity diagnosed with T2D
T2D - All of Us	Hemoglobin A1c measurement
T2D - All of Us	Serum glucose measurement
T2D - All of Us	Urine microalbumin measurement
T2D - All of Us	Serum creatinine measurement
T2D - All of Us	Lipid panel
T2D - All of Us	Blood urea nitrogen measurement
T2D - All of Us	Serum potassium measurement
T2D - All of Us	Serum sodium measurement
T2D - All of Us	Serum chloride measurement
T2D - All of Us	Serum bicarbonate measurement
T2D - All of Us	Serum calcium measurement
T2D - All of Us	Serum magnesium measurement
T2D - All of Us	Serum albumin measurement
T2D - All of Us	Serum bilirubin measurement
T2D - All of Us	Serum alkaline phosphatase measurement
T2D - All of Us	Serum aspartate aminotransferase measurement

Cohort	Test Name
T2D - All of Us	Serum alanine aminotransferase measurement
T2D - All of Us	Serum lactate dehydrogenase measurement
T2D - All of Us	Serum uric acid measurement
T2D - All of Us	Serum triglycerides measurement
T2D - All of Us	Serum high density lipoprotein measurement
T2D - All of Us	Serum low density lipoprotein measurement
T2D - All of Us	Serum phosphate measurement
T2D - All of Us	Serum cholesterol measurement

Supplementary Table S6: Test suggestion validation prompts

Validation of test suggestion tables

```
For the following test, please evaluate the reference value by searching for the
correct reference value.
It appears as the expected value or range. Please make sure that the reference value
is correct.
If there is no available data for {region}, please provide the reference value for
the whole country, continent or global statistics.
If it is incorrect, please provide the correct value. If you are unsure, please
indicate that as well.
For disease-related tests, the reference value represents the prevalence of that
disease among patients with {diagnosis} in {region}.
Demographics - unless diagnosed is 'True', the percentage is relative to the general
population in {region}.
For those diagnosed with {diagnosis} in {region}, diagnosed flag will be 'True', and
the percentage is the percentage of {diagnosis} patients among the evaluated

statistic (e.g, percentage of diagnosed women among all women or percentage of
patients prescribed a drug among all patients with {diagnosis}).

The test suggestion is as follows:

Column names: {list(row.index)}
Values: {row.values}

diagnosed: {diagnosed}

Please provide your feedback in the following format:
1. Test description: [your answer]
2. Correct reference value: [your answer]
3. Provided reference value: [your answer]
4. Feedback: [your answer - Correct or Incorrect or Unsure]
5. Fuction recommendation: [your answer - Fix (if incorrect), No fix (if correct), Remove
```

```
If according to your feedback the test suggestion needs fixing due to reference
value correction, please provide it betweeen triple backticks (```csv). use a
vertical line | as separator.
do not change anything but the reference value and the comparison type, if needed.
Strictly stick to the original field names and order. To the field names add 'fixed'
and 'remove'.
Set 'fixed' to be 'True' to the test suggestion if the function needs to be fixed.
Set it to be 'False' otherwise.
Make sure the fixed value corrersponds with the correct value you found.
If the expected value is a range and not a single value, please fill the range_high
and range_low columns with the correct values, and remove the incorrect value. In
that case - change the comparison_type to 'range'.
Please make sure the numbers are in the correct location (minimum and maximum or
high and low).
The comparison_type can also be set to 'less_than' or 'greater_than', in there is a
minimum or a maximum only value, respectively.
If you recommended removing the test, please set the 'remove' column in the test
suggestion to be 'True'. No need to fix it in that case.
If the column needs to be fixed, fix it, set 'fixed' to be 'True' and set 'remove'
column to be 'False'.


If a test repeats - in terms of 'description' and 'expected_value' or range columns,
please set the 'remove' column to be 'True' for the repeated test suggestion.
Please add the following references (including urls) to the 'references' column in
the corrected csv. Add all the values in the 'references' column in parentheses "".

     {replies[-1][ref_loc:]}

Return the test anyway, even if it is correct, with the additional columns (both set
to 'False', if fix or removal isn't suggested).
```

Validation of statistical values

```
For the following statistics, please evaluate the reference value by searching for
the correct reference value.
It appears as the expected value or range. Please make sure that the reference value
is correct.
If there is no available data for {region}, please provide the reference value for
the whole country, continent or global statistics.
If it is incorrect, please provide the correct value. If you are unsure, please
indicate that as well.

The statistics are for {diagnosis} in {region}:

{stats}

Please provide your feedback in the following format for each statistic:
1. Statistic: [your answer]
2. Correct reference value: [your answer]
3. Provided reference value: [your answer]
4. Feedback: [your answer - Correct or Incorrect or Unsure]
5. Recommendation: [your answer - Fix (if incorrect), No fix (if correct), Remove
(if unsure)]
```

```
Please provide the modified statistics in a similar format as the original, betweeen
triple backticks (‘‘‘csv).
use a vertical line | as separator.
If according to your feedback the statitic suggestion needs fixing due to reference
value correction, please correct it.
do not change anything but the reference value.
Strictly stick to the original field names and order.
Make sure the fixed value corrersponds with the correct value you found.
If you recommended removing the statistic, replace the relevant statistic with
’np.nan’.
Please add the following references (including urls) to the ’reference’ column in
the corrected csv. Add all the values in the ’reference’ column in parentheses "".

{replies[-1][ref_loc:]}
```

```
For the following statistics, please evaluate the reference value by searching for
the correct reference value.
It appears as the expected value or range. Please make sure that the reference value
is correct.
If there is no available data for {region}, please provide the reference value for
the whole country, continent or global statistics.
If it is incorrect, please provide the correct value. If you are unsure, please
indicate that as well.

The statistics are for {diagnosis} in {region}:

{age}

Please provide your feedback in the following format for each statistic:
1. Statistic: [your answer]
2. Correct reference value: [your answer]
3. Provided reference value: [your answer]
4. Feedback: [your answer - Correct or Incorrect or Unsure]
5. Recommendation: [your answer - Fix (if incorrect), No fix (if correct), Remove
(if unsure)]
```

```
Please provide the modified statistics in a similar format as the original, betweeen
triple backticks (‘‘‘csv).
use a vertical line | as separator.
If according to your feedback the statitic suggestion needs fixing due to reference
value correction, please correct it.
do not change anything but the reference value.
Strictly stick to the original field names and order.
Make sure the fixed value corrersponds with the correct value you found.
If you recommended removing the statistic, replace the relevant statistic with
’np.nan’.
Please add the following references (including urls) to the ’reference’ column in
the corrected csv. Add all the values in the ’reference’ column in parentheses "".

{replies[-1][ref_loc:]}
```

	Valid References	Total Tests	Accurate	Inaccurate	Incorrect	Qualitative	Irrelevant
**T2D**	92.9%	57	62.5%	17.9%	19.6%	0%	0%
**CKD**	49.2%	60	65.0%	16.7%	8.3%	3.4%	5.0%
**HTN**	98.3%	61	68.9%	6.6%	14.8%	4.9%	3.3%
**CHF**	39.5%	44	47.7%	25.0%	15.9%	2.3%	6.8%

Supplementary Table S7: Test quality validation statistics when tests that evaluate prevalence of drug usage are disregarded.

		Population Characteristics
n		410,361
**Gender, n (%)**	Female	245,601 (59.8)
	Male	152,142 (37.1)
	No matching concept	97 (0.0)
	Other / Skipped	12,521 (3.1)
**Race, n (%)**	Another single population	2,987 (0.7)
	Asian	14,162 (3.5)
	Black or African American	78,321 (19.1)
	Prefer not to answer	2,537 (0.6)
	More than one population	7,720 (1.9)
	None indicated	64,614 (15.7)
	None of these	4,351 (1.1)
	Skip	9,054 (2.2)
	White	226,615 (55.2)
**Ethnicity, n (%)**	Hispanic or Latino	73,945 (18.0)
	No matching concept	97 (0.0)
	Not Hispanic or Latino	320,377 (78.1)
	Prefer not to answer	2,537 (0.6)
	Skip	9,054 (2.2)
	None of these	4,351 (1.1)
**Sex at Birth, n (%)**	Female	248,130 (60.5)
	Male	153,582 (37.4)
	No matching concept	4,283 (1.0)
	Other / Skipped	4,366 (1.1)
**Age, mean (SD)**		56.0 (16.8)

Supplementary Table S8: All of Us descriptive statistics.

Test	T2D	CHF	CKD	HTN
**Total Functions**	60	55	67	73
Total Functions Passed	6	4	2	2
Total Functions Failed	54	51	65	71
Total Functions Correct	35	23	40	43
Total Functions Non-correct	24	31	26	29
Correct and Passed	1	3	0	0
Correct and Failed	34	20	40	43
Non-correct and Passed	5	1	2	2
Non-correct and Failed	19	30	24	27
**Metadata Total**	1	1	1	1
Metadata pass	0	0	0	0
Metadata fail	1	1	1	1
**Subpopulation Total**	19	20	22	22
Subpopulation Correct	6	3	9	9
Subpopulation Non-correct	13	17	13	13
Subpopulation Passed	2	0	0	1
Subpopulation Failed	17	20	22	21
Subpopulation Correct and Passed	0	0	0	0
Subpopulation Correct and Failed	6	3	9	9
Subpopulation Non-correct and Passed	2	0	0	1
Subpopulation Non-correct and Failed	11	17	13	12
**Distribution Total**	40	34	44	50
Distribution Passed	4	4	2	1
Distribution Failed	36	30	42	49
Distribution Correct	29	20	31	32
Distribution Non-correct	11	14	13	18
Distribution Correct and Passed	1	3	0	0
Distribution Correct and Failed	28	17	31	32
Distribution Non-correct and Passed	3	1	2	1
Distribution Non-correct and Failed	8	13	11	17
**Total Non-correct Functions**	24	31	26	29
Total inaccurate	10	12	12	5
Inaccurate and Passed	3	1	2	1
Inaccurate and Failed	7	11	10	4
Total incorrect	12	11	8	19
Incorrect and Passed	1	0	0	1
Incorrect and Failed	11	11	8	18
Total irrelevant	2	8	6	5
Irrelevant and Passed	1	0	0	0
Irrelevant and Failed	1	8	6	5

Supplementary Table S9: Full results describing MDPT performance on the four datasets, in terms of pass/fail and reference correctness.

Supplementary Table S10: Failed Tests with Zero Prevalence

Cohort	Subcategory	Test Name
CHF	Demographics (Race/Ethnicity/Gender)	Ethnicity of the patient Asian
CHF	Demographics (Race/Ethnicity/Gender)	Ethnicity of the patient Black African American
CHF	Demographics (Race/Ethnicity/Gender)	Ethnicity of the patient Hispanic Latino
CHF	Demographics (Race/Ethnicity/Gender)	Ethnicity of the patient Other
CHF	Demographics (Race/Ethnicity/Gender)	Gender of the patient Male
CHF	Medications / Drugs	Bisoprolol
CHF	Medications / Drugs	Carvedilol
CHF	Medications / Drugs	Digoxin
CHF	Medications / Drugs	Eplerenone
CHF	Medications / Drugs	Furosemide
CHF	Medications / Drugs	Ivabradine
CHF	Medications / Drugs	Lisinopril
CHF	Medications / Drugs	Sacubitril_Valsartan
CHF	Medications / Drugs	Spironolactone
CHF	Medications / Drugs	Torsemide
CHF	Medications / Drugs	Valsartan
CHF	Other / General Checks	check_incidence
CHF	Other / General Checks	check_prevalence
CKD	Demographics (Race/Ethnicity/Gender)	Race American Indian or Alaska Native
CKD	Demographics (Race/Ethnicity/Gender)	Race Asian
CKD	Demographics (Race/Ethnicity/Gender)	Race Native Hawaiian or Other Pacific Islander
CKD	Diagnoses / Conditions	Coronary Artery Disease diagnosis codes among diagnosed patients
CKD	Diagnoses / Conditions	Depression diagnosis codes among diagnosed patients
CKD	Diagnoses / Conditions	Diabetes Mellitus with complication diagnosis codes among diagnosed patients

Continued on next page

Supplementary Table S10: Failed Tests with Zero Prevalence

Cohort	Subcategory	Test Name
CKD	Diagnoses / Conditions	Diabetes Mellitus without complication diagnosis codes among diagnosed patients
CKD	Diagnoses / Conditions	Gout diagnosis codes among diagnosed patients
CKD	Diagnoses / Conditions	Hyperparathyroidism diagnosis codes among diagnosed patients
CKD	Diagnoses / Conditions	Hypertension diagnosis codes among diagnosed patients
CKD	Diagnoses / Conditions	Liver Disease diagnosis codes among diagnosed patients
CKD	Diagnoses / Conditions	Osteopenia diagnosis codes among diagnosed patients
CKD	Diagnoses / Conditions	Osteoporosis diagnosis codes among diagnosed patients
CKD	Diagnoses / Conditions	Sleep Apnea diagnosis codes among diagnosed patients
CKD	Medications / Drugs	Amlodipine
CKD	Medications / Drugs	Calcitriol
CKD	Medications / Drugs	Cinacalcet
CKD	Medications / Drugs	Erythropoietin
CKD	Medications / Drugs	Furosemide
CKD	Medications / Drugs	Lisinopril
CKD	Medications / Drugs	Metoprolol
HTN	Demographics (Race/Ethnicity/Gender)	Hispanic or Latino ethnicity
HTN	Demographics (Race/Ethnicity/Gender)	Native Hawaiian or Other Pacific Islander race
HTN	Demographics (Race/Ethnicity/Gender)	Not Hispanic or Latino ethnicity
HTN	Demographics (Race/Ethnicity/Gender)	Other race
HTN	Diagnoses / Conditions	Anemia diagnosis code among diagnosed patients
HTN	Diagnoses / Conditions	Atrial fibrillation and related conditions diagnosis code among diagnosed patients

Continued on next page

Supplementary Table S10: Failed Tests with Zero Prevalence

Cohort	Subcategory	Test Name
HTN	Diagnoses / Conditions	Chronic obstructive pulmonary disease diagnosis code among diagnosed patients
HTN	Diagnoses / Conditions	Depression diagnosis code among diagnosed patients
HTN	Diagnoses / Conditions	Heart failure diagnosis code among diagnosed patients
HTN	Diagnoses / Conditions	Hyperlipidemia diagnosis code among diagnosed patients
HTN	Diagnoses / Conditions	Peripheral vascular disease diagnosis code among diagnosed patients
HTN	Diagnoses / Conditions	Sleep apnea and related conditions diagnosis code among diagnosed patients
HTN	Diagnoses / Conditions	Stroke and related conditions diagnosis code among diagnosed patients
HTN	Medications / Drugs	Amlodipine
HTN	Medications / Drugs	Atenolol
HTN	Medications / Drugs	Carvedilol
HTN	Medications / Drugs	Enalapril
HTN	Medications / Drugs	Furosemide
HTN	Medications / Drugs	Hydrochlorothiazide
HTN	Medications / Drugs	Lisinopril
HTN	Medications / Drugs	Losartan
HTN	Medications / Drugs	Metoprolol
HTN	Medications / Drugs	Valsartan
T2D	Demographics (Race/Ethnicity/Gender)	American Indian or Alaska Native
T2D	Demographics (Race/Ethnicity/Gender)	Asian
T2D	Demographics (Race/Ethnicity/Gender)	Native Hawaiian or Other Pacific Islander
T2D	Diagnoses / Conditions	Anemia diagnosis code among diagnosed patients
T2D	Diagnoses / Conditions	Coronary artery disease diagnosis code among diagnosed patients

Continued on next page

Supplementary Table S10: Failed Tests with Zero Prevalence

Cohort	Subcategory	Test Name
T2D	Diagnoses / Conditions	Depression diagnosis code among diagnosed patients
T2D	Diagnoses / Conditions	Hypertension diagnosis code among diagnosed patients
T2D	Diagnoses / Conditions	Osteoarthritis diagnosis code among diagnosed patients
T2D	Diagnoses / Conditions	Sleep apnea diagnosis code among diagnosed patients
T2D	Diagnoses / Conditions	Stroke diagnosis code among diagnosed patients
T2D	Medications / Drugs	Metformin
T2D	Medications / Drugs	Sitagliptin
T2D	Other / General Checks	Other

Supplementary Table S11: Sensitivity Analysis Results

Cohort	Number of Tests	SMD	Proportional Tolerance	Fail Number (All)	Fail Rate (All)	Fail Number (Correct)	Fail Rate (Correct)
midrule							
Congestive Heart Failure (MIMIC-III)	55	0.2	0.10	53	0.964	22	0.957
			0.15	51	0.927	20	0.870
			0.20	50	0.909	19	0.826
			0.25	50	0.909	19	0.826
			0.30	50	0.909	19	0.826
		0.5	0.10	53	0.964	22	0.957
			0.15	51	0.927	20	0.870
			0.20	50	0.909	19	0.826
			0.25	50	0.909	19	0.826
			0.30	50	0.909	19	0.826
		0.8	0.10	53	0.964	22	0.957
			0.15	51	0.927	20	0.870
			0.20	50	0.909	19	0.826
			0.25	50	0.909	19	0.826
			0.30	50	0.909	19	0.826

Continued on next page

Supplementary Table S11: Sensitivity Analysis Results

Cohort	Number of Tests	SMD	Proportional Tolerance	Fail Number (All)	Fail Rate (All)	Fail Number (Correct)	Fail Rate (Correct)
Hypertension (SyntheticMass)	73	0.2	0.10	72	0.986	43	1.000
			0.15	71	0.973	43	1.000
			0.20	70	0.959	43	1.000
			0.25	70	0.959	43	1.000
			0.30	70	0.959	43	1.000
		0.5	0.10	72	0.986	43	1.000
			0.15	71	0.973	43	1.000
			0.20	69	0.945	42	0.977
			0.25	68	0.932	41	0.953
			0.30	67	0.918	40	0.930
		0.8	0.10	72	0.986	43	1.000
			0.15	71	0.973	43	1.000
			0.20	69	0.945	42	0.977
			0.25	68	0.932	41	0.953
			0.30	67	0.918	40	0.930
Type 2 Diabetes (All of Us)	60	0.2	0.10	54	0.900	34	0.971
			0.15	54	0.900	34	0.971
			0.20	52	0.867	33	0.943
			0.25	52	0.867	33	0.943
			0.30	52	0.867	33	0.943
		0.5	0.10	54	0.900	34	0.971
			0.15	54	0.900	34	0.971
			0.20	52	0.867	33	0.943
			0.25	52	0.867	33	0.943
			0.30	50	0.833	31	0.886
		0.8	0.10	54	0.900	34	0.971
			0.15	54	0.900	34	0.971
			0.20	52	0.867	33	0.943
			0.25	52	0.867	33	0.943
			0.30	50	0.833	31	0.886
Chronic Kidney Disease (All of Us)	67	0.2	0.10	65	0.970	40	1.000
			0.15	65	0.970	40	1.000
			0.20	64	0.955	39	0.975
			0.25	64	0.955	39	0.975

Continued on next page

Supplementary Table S11: Sensitivity Analysis Results

Cohort	Number of Tests	SMD	Proportional Tolerance	Fail Number (All)	Fail Rate (All)	Fail Number (Correct)	Fail Rate (Correct)
			0.30	64	0.955	39	0.975
		0.5	0.10	65	0.970	40	1.000
			0.15	65	0.970	40	1.000
			0.20	64	0.955	39	0.975
			0.25	64	0.955	39	0.975
			0.30	62	0.925	37	0.925
		0.8	0.10	65	0.970	40	1.000
			0.15	65	0.970	40	1.000
			0.20	64	0.955	39	0.975
			0.25	64	0.955	39	0.975
			0.30	62	0.925	37	0.925

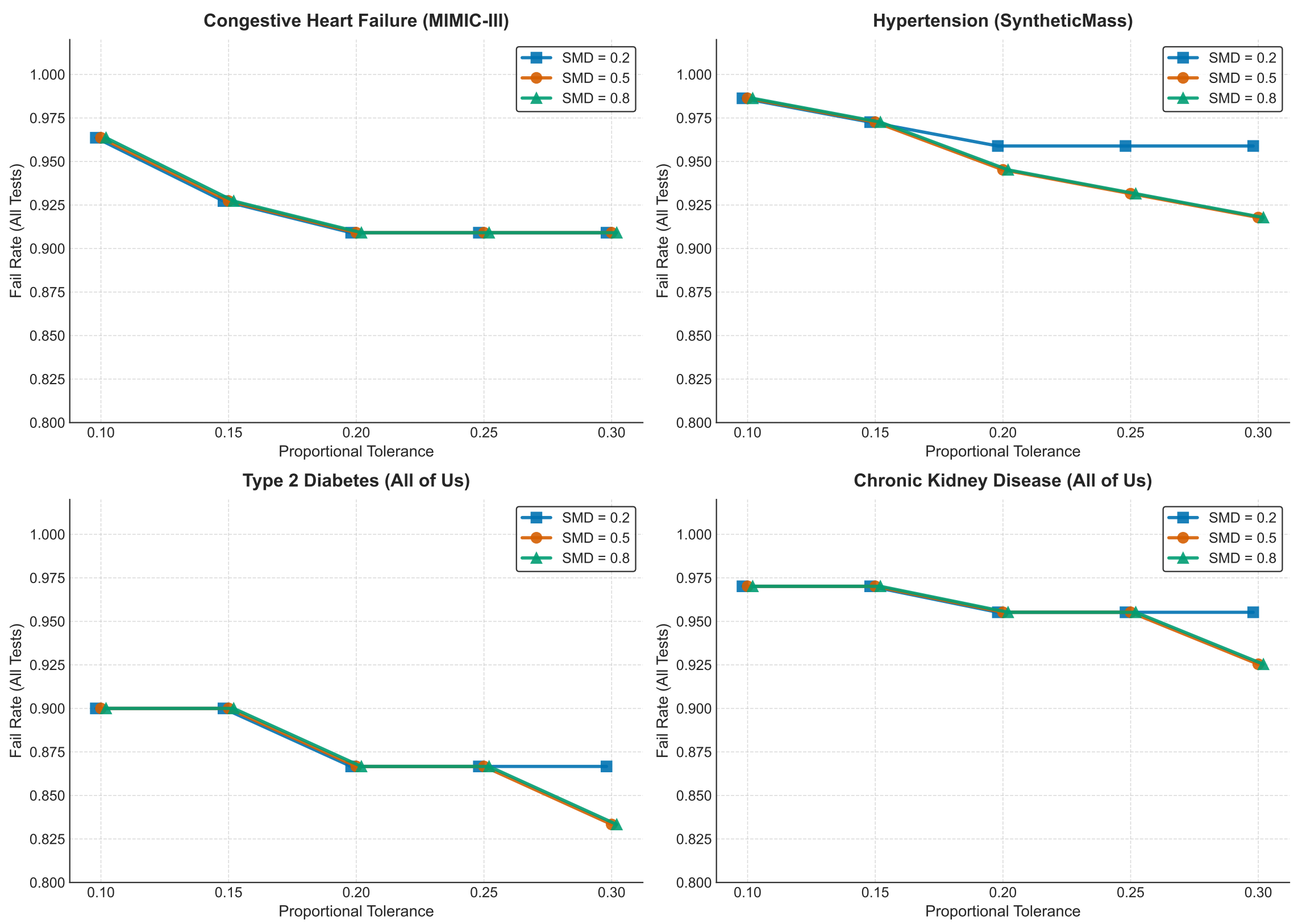

Supplementary Figure S3: **Failure rates across statistical threshold combinations for all generated tests.** Failure rates for each cohort are shown across proportional tolerance thresholds of 10%, 15%, 20%, 25%, and 30%, with separate curves for SMD cutoffs of 0.2, 0.5, and 0.8, considering all generated tests.

References

1. Mirzadeh I, Alizadeh K, Shahrokhi H, Tuzel O, Bengio S, and Farajtabar M. GSM-Symbolic: Understanding the Limitations of Mathematical Reasoning in Large Language Models. arXiv:2410.05229 [cs] version: 1. 2024. DOI: 10.48550/arXiv.2410.05229.
2. Narayan A, Chami I, Orr L, Arora S, and Ré C. Can Foundation Models Wrangle Your Data? arXiv:2205.09911 [cs]. 2022. DOI: 10.48550/arXiv.2205.09911.
