# Supplementary material for: A generative approach for semantic auditing of electronic health records
Source: NPJ Digit Med. 2026 May 30;9:666. doi: 10.1038/s41746-026-02809-w (PMC13522542; doi:10.1038/s41746-026-02809-w)
Supplement: Supplementary file 2 [file 41746_2026_2809_MOESM2_ESM.pdf]

Type 2 diabetes in US - Data Quality Evaluation Report

January 8, 2026

1 Demography and Diagnosis Tests

Test name	Result	Expected value	Actual value	SMD	ratio
American Indian or Alaska Native	Fail	1.1	0.00	-0.15	0.00
Anemia diagnosis diagnosed	Fail	17.8	0.00	-0.66	0.00
Asian	Fail	6.2	0.00	-0.36	0.00
Black or African American	Fail	13.6	19.09	0.15	1.40
Black or African American diagnosed	Pass	18.0	16.39	-0.04	0.91
Chronic kidney disease diagnosis diagnosed	No Reference				
Coronary artery disease diagnosis diagnosed	No Reference				
Depression diagnosis diagnosed	Fail	19.0	0.00	-0.68	0.00
Fatty liver disease diagnosis diagnosed	No Reference				
Female	Fail	50.8	59.85	0.18	1.18
Hispanic or Latino	Pass	18.9	18.02	-0.02	0.95
Hispanic or Latino diagnosed	Fail	17.0	14.38	-0.07	0.85
Hyperlipidemia diagnosis diagnosed	Pass	70.0	74.32	0.10	1.06
Hypertension diagnosis diagnosed	Fail	70.0	0.00	-2.16	0.00
Male	Fail	49.5	37.08	-0.25	0.75
Male diagnosed	Fail	51.0	13.40	-0.88	0.26
Native Hawaiian or Other Pacific Islander	Fail	0.2	0.00	-0.06	0.00
Not Hispanic or Latino	Pass	81.3	78.07	-0.08	0.96
Not Hispanic or Latino diagnosed	Fail	66.0	11.17	-1.36	0.17
Osteoarthritis diagnosis diagnosed	Fail	29.5	0.00	-0.91	0.00
Other	Fail	6.2	0.00	-0.36	0.00
Peripheral vascular disease diagnosis diagnosed	No Reference				
Retinopathy diagnosis diagnosed	Fail	28.5	0.50	-0.87	0.02
Sleep apnea diagnosis diagnosed	Fail	18.0	0.00	-0.66	0.00
Stroke diagnosis diagnosed	Fail	20.0	0.00	-0.71	0.00
White	Fail	75.8	55.22	-0.44	0.73
White diagnosed	Fail	58.0	9.69	-1.19	0.17
check incidence	Fail	1.4	5.86	0.24	4.19
check prevalence	Pass	11.3	11.80	0.02	1.04
check data types	Fail				

2 Drug Tests

Test name	Result	Expected value	Actual value	SMD	ratio
A foreign key to a standard concept identifier for the drug concept 1	Fail	48.6	0.00	-1.38	0.00
A foreign key to a standard concept identifier for the drug concept 2	No Reference				
A foreign key to a standard concept identifier for the drug concept 3	Pass	48.6	0.00		
check data types	Fail				

3 Measurement Tests

Test name	Result	Expected value	Actual value	SMD	ratio
Blood urea nitrogen measurement	Fail	95%	0.00		
Hemoglobin A1c measurement	Fail	95%	60.64		
Lipid panel	Fail	95%	0.00		
Serum alanine aminotransferase measurement	Fail	95%	0.00		
Serum albumin measurement	Fail	95%	0.00		
Serum alkaline phosphatase measurement	Fail	95%	0.00		
Serum aspartate aminotransferase measurement	Fail	95%	0.00		
Serum aspartate aminotransferase measurement 1	Fail	95%	0.00		
Serum bicarbonate measurement	Fail	95%	0.00		
Serum bilirubin measurement	Fail	95%	0.00		
Serum calcium measurement	Fail	95%	0.00		
Serum chloride measurement	Fail	95%	10.12		
Serum cholesterol measurement	Fail	95%	7.86		
Serum creatinine measurement	Fail	95%	0.00		
Serum creatinine measurement 1	Fail	95%	0.00		
Serum glucose measurement	Fail	95%	85.25		
Serum high density lipoprotein measurement	Fail	95%	48.28		
Serum lactate dehydrogenase measurement	Fail	95%	0.00		
Serum low density lipoprotein measurement	Fail	95%	52.84		
Serum magnesium measurement	Fail	95%	0.00		
Serum phosphate measurement	Fail	95%	0.00		
Serum potassium measurement	Fail	95%	0.00		
Serum potassium measurement 1	Fail	95%	0.00		
Serum sodium measurement	Fail	95%	0.00		
Serum triglycerides measurement	Fail	95%	0.00		
Serum uric acid measurement	Fail	95%	0.00		
Serum uric acid measurement 1	Fail	95%	0.00		
Urine microalbumin measurement	Fail	30.0	74.61		
Urine microalbumin measurement 1	Fail	95%	74.66		
check data types	Fail				
